# The *Vinca minor* genome highlights conserved evolutionary traits in monoterpene indole alkaloid synthesis

**DOI:** 10.1093/g3journal/jkac268

**Published:** 2022-10-06

**Authors:** Emily Amor Stander, Clément Cuello, Caroline Birer-Williams, Natalja Kulagina, Hans J Jansen, Ines Carqueijeiro, Louis-Valentin Méteignier, Valentin Vergès, Audrey Oudin, Nicolas Papon, Ron P Dirks, Michael Krogh Jensen, Sarah Ellen O’Connor, Thomas Dugé de Bernonville, Sébastien Besseau, Vincent Courdavault

**Affiliations:** Biomolécules et Biotechnologies Végétales, EA2106, Université de Tours, 37200 Tours, France; Biomolécules et Biotechnologies Végétales, EA2106, Université de Tours, 37200 Tours, France; Biomolécules et Biotechnologies Végétales, EA2106, Université de Tours, 37200 Tours, France; Biomolécules et Biotechnologies Végétales, EA2106, Université de Tours, 37200 Tours, France; Future Genomics Technologies, 2333 BE Leiden, The Netherlands; Biomolécules et Biotechnologies Végétales, EA2106, Université de Tours, 37200 Tours, France; Biomolécules et Biotechnologies Végétales, EA2106, Université de Tours, 37200 Tours, France; Biomolécules et Biotechnologies Végétales, EA2106, Université de Tours, 37200 Tours, France; Biomolécules et Biotechnologies Végétales, EA2106, Université de Tours, 37200 Tours, France; Univ Angers, Univ Brest, IRF, SFR ICAT, F-49000 Angers, France; Future Genomics Technologies, 2333 BE Leiden, The Netherlands; Novo Nordisk Foundation Center for Biosustainability, Technical University of Denmark, 2800 Kongens Lyngby, Denmark; Department of Natural Product Biosynthesis, Max Planck Institute for Chemical Ecology, Jena 07745, Germany; Biomolécules et Biotechnologies Végétales, EA2106, Université de Tours, 37200 Tours, France; Biomolécules et Biotechnologies Végétales, EA2106, Université de Tours, 37200 Tours, France; Biomolécules et Biotechnologies Végétales, EA2106, Université de Tours, 37200 Tours, France

**Keywords:** *Vinca minor*, lesser periwinkle, genome, alkaloids, vincadifformine

## Abstract

*Vinca minor*, *also* known as the lesser periwinkle, is a well-known species from the *Apocynaceae*, native to central and southern Europe. This plant synthesizes monoterpene indole alkaloids, which are a class of specialized metabolites displaying a wide range of bioactive- and pharmacologically important properties. Within the almost 50 monoterpene indole alkaloids it produces, *V. minor* mainly accumulates vincamine, which is commercially used as a nootropic. Using a combination of Oxford Nanopore Technologies long read- and Illumina short-read sequencing, a 679,098 Mb *V. minor* genome was assembled into 296 scaffolds with an N50 scaffold length of 6 Mb, and encoding 29,624 genes. These genes were functionally annotated and used in a comparative genomic analysis to establish gene families and to investigate gene family expansion and contraction across the phylogenetic tree. Furthermore, homology-based monoterpene indole alkaloid gene predictions together with a metabolic analysis across 4 different *V. minor* tissue types guided the identification of candidate monoterpene indole alkaloid genes. These candidates were finally used to identify monoterpene indole alkaloid gene clusters, which combined with synteny analysis allowed for the discovery of a functionally validated vincadifformine-16-hydroxylase, reinforcing the potential of this dataset for monoterpene indole alkaloids gene discovery. It is expected that access to these resources will facilitate the elucidation of unknown monoterpene indole alkaloid biosynthetic routes with the potential of transferring these pathways to heterologous expression systems for large-scale monoterpene indole alkaloid production.

## Introduction

**Fig. 1.**
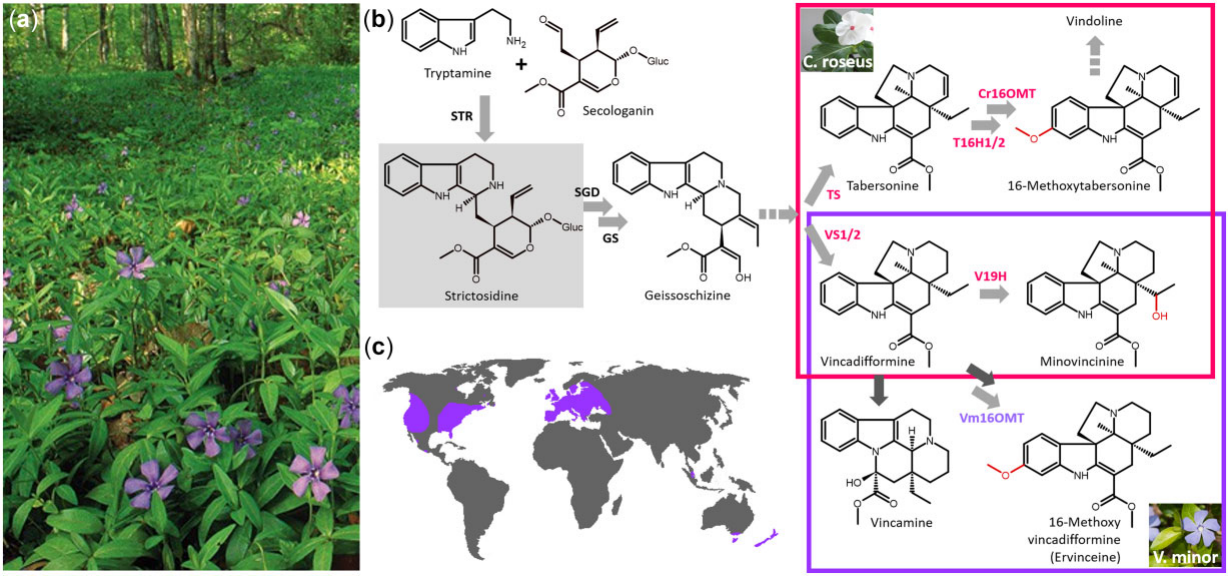
Overview of *Vinca minor*. a) The so-called lesser periwinkle, this MIA accumulating *Apocynaceae* is a ground cover undergrowth plant, native to central Europe. b) Simplified biosynthesis pathway leading to several important eburnamine/aspidosperma-type MIAs produced in *V. minor* (lower box) and *C. roseus* (upper box). The central MIA strictosidine is highlighted in grey. Light gray and dark gray arrows indicate characterized and uncharacterized enzymatic steps respectively, whereas dash arrows correspond to multiple enzymatic steps. Enzymes characterized in *V. minor* and *C. roseus* are annotated in purple and pink respectively. *STR*, strictosidine synthase; *SGD*, strictosidine β-d-glucosidase; *GS*, geissoschizine synthase; *TS*, tabersonine synthase; *T16H*, tabersonine 16-hydroxylase; *Cr16OMT*, 16-hydroxytabersonine 16-*O*-methyltransferase; *VS*, vincadifformine synthase; *V19H*, vincadifformine 19-hydroxylase; *Vm16OMT*, 16-hydroxyvincadifformine 16-*O*-methyltransferase. c) Reported geographical distribution of *V. minor*.

Monoterpene indole alkaloids (MIAs) are a class of specialized (also known as secondary) metabolites produced by plants from the *Gentianale* order families, including *Gelsemiaceae*, *Apocynaceae, Loganiaceae*, and *Rubiaceae* but also by a few *Nyssaceae* from the *Cornale* order (O’Connor and Maresh 2006). MIAs display a broad spectrum of bioactive properties and thus belong to the plethora of chemical arsenals that plants evolved to cope with environmental pressure and notably abiotic and biotic attacks (Dugé de Bernonville *et al.* 2015). These biological activities make MIAs attractive pharmaceuticals of high economic value, as illustrated with ajmaline from *Rauwolfia* species (*Apocynaceae*) prescribed for the treatment of arrhythmia as well as vinblastine and vincristine from *Catharanthus roseus* (*Apocynaceae*) that are potent anticancer compounds (O’Connor and Maresh 2006). The lesser periwinkle, *Vinca minor*, also belongs to the *Apocynaceae* family and accumulates more than 50 MIAs (Proksa and Grossmann 1991; D’Amelio Sr *et al.* 2012; Hasa *et al.* 2013; Vrabec *et al.* 2022) including aspidosperma-type MIAs such as vincadifformine and eburnamine-type MIAs such as vincamine which is commercially used as a vasodilator together with its semisynthetic derivative vinpocetine (Vas and Gulyás 2005) (Fig. 1, a–c).

MIAs result from long and complex biosynthetic pathways sharing high levels of similarity among species (Fig. 1a). With only a few exceptions, all MIAs originate from strictosidine that is produced by the condensation of secologanin and tryptamine, the monoterpene, and indole MIA precursors, respectively. The subsequent strictosidine decorations direct MIA synthesis toward the almost 3,000 described MIAs. To date, most of our knowledge on these manifold reactions arises from *C. roseus* (Courdavault *et al.* 2014; Kulagina *et al.* 2022). This notably led to the characterization of the biosynthetic routes towards major MIAs such as catharanthine and tabersonine (Tatsis *et al.* 2017; Caputi *et al.* 2018; Qu *et al.* 2019). Interestingly, tabersonine can also be diverted to the famous vindoline through a 7-step pathway initiated by a couple of tabersonine 16-hydroxylases (*T16H*; Besseau *et al.* 2013) and an associated 16-hydroxytabersonine 16-*O*-methyltransferase (*16OMT*) (Levac *et al.* 2008). In *V. minor*, while vincamine is highly accumulated (Proksa and Grossmann 1991), its biosynthesis remains mostly unknown. However, it has been suggested that this compound derives from vincadifformine whose synthesis relies on 2 vincadifformine synthases (*VS*) as described in *C. roseus* (Williams *et al.* 2019; Caputi *et al.* 2020). Interestingly, a P450 from *C. roseus* has been proposed to catalyze a cyclization of tabersonine/vincadifformine leading to the synthesis of an eburnamine-vincamine skeleton product but no ortholog from the lesser periwinkle has been identified to date (Kellner *et al.* 2015a). Overall, only a few MIA biosynthetic genes have been identified and characterized in *V. minor* including a picrinine N-methyltransferase, (*VmPiNMT*; Levac *et al.* 2016), a vincamine/vincadifformine ATP binding cassette transporter (*VmTPT2*/*VmABCG1*; Demessie *et al.* 2017), and a vincadifformine 16-*O*-methyltransferase (*Vm16OMT*; Stander *et al.* 2020).

While almost all MIA biosynthetic gene identifications have resulted from transcriptomics and gene co-expression analyses to date (Dugé de Bernonville *et al.* 2020), the ever-growing access to plant genome sequences opens new perspectives towards MIA pathway elucidation (Stander *et al.* 2022). To date, 7 nuclear genomes of MIA producing species have been sequenced thus providing new insights into the specific MIA synthesis of each plant species (Table 1). Here, we report the genome assembly, annotation, and analysis of *V. minor* combined with the identification and functional validation of vincadifformine 16-hydroxylase. Such a genomic resource will potentially pave the way for future MIA biosynthetic gene identification in this prominent medicinal plant species.

**Table 1.** Main features of nuclear genomes from MIA producing plant species.

Plant	Family	Assembly size	No. of scaffolds/ pseudo-chromosomes	N50 scaffold (Mb)	Reference
*Mitragyna speciosa*	*Rubiaceae*	1.1 Gb	17,031	1	Brose *et al.* (2021)
*Gelsemium sempervirens*	*Gelsemiaceae*	244 Mb	3,352	0.41	Franke *et al.* (2019)
*Catharanthus roseus V2*	*Apocynaceae*	541 Mb	2,090	2.57	Franke *et al.* (2019)
*Camptotheca acuminata V3*	*Cornales*	414,95 Mb	21	18.28	Kang *et al.* (2021)
*Ophiorrhiza pumila*	*Rubiaceae*	439.90 Mb	11	42.83	Rai *et al.* (2021)
*Rhazya stricta*	*Apocynaceae*	274.0 Mb	980	5.5 Mb	Sabir *et al.* (2016)
*Neolamarckia cadamba*	*Rubiaceae*	744.5 Mb	22	29.20	Zhao *et al.* (2022)

## Materials and methods

### Sample collection

Wild-growing *V. minor* plants were collected in Tours, France (4721011.400 N 042008.200 E) for direct DNA extraction and MIA quantification.

### Chemicals

Vincadifformine was purchased from Biosynth Carbosynth (UK). 16-hydroxyvincadifformine was produced by a yeast strain expressing *C. roseusT16H2* via 16-hydroxylation of vincadifformine (Stander *et al.* 2020).

### DNA extraction and sequencing

Nuclei were first isolated from young leaves following the procedure described in Workman *et al.* (2018). High-molecular weight DNA was extracted from *V. minor* leaves using the Nanobind HMW DNA Extraction Circulomics kit (Circulomics Inc., Baltimore, MD, USA) as per manufacturer’s instructions. For Illumina sequencing, a sequencing library was constructed using the TruSeq DNA PCR-Free Library Prep kit (Illumina, San Diego, USA) and sequenced in paired-end mode (2 × 100 bp) by Eurofins Genomics (Les Ulis, France) using Illumina Novaseq 6000 technology. Oxford Nanopore Technologies (ONT) library construction and sequencing were performed at Future Genomics Technologies (Leiden, The Netherlands). Library was constructed from approximately 1 µg of high-molecular weight DNA using Ligation sequencing kit (SQK-LSK109, Oxford Nanopore Technologies Ltd) and sequenced on a Nanopore PromethION flowcell (FLO-PRO002, Oxford Nanopore Technologies Ltd) with the guppy version 3.2.6 high-accuracy basecaller.

The estimated genome length and heterozygosity were determined by calculating the *k*-mer frequencies (*k* = 21) from Illumina short-reads with Jellyfish (v.2.3.0, Marçais and Kingsford 2011) using the following parameters: jellyfish count -C -m 21 -s 1000000000 -t 10. The resulting k-mer count histogram was imported into Genomescope (Vurture *et al.* 2017).

### De novo genome assembly

The *V. mino*r genome was assembled by Future Genomics Technologies (Leiden, The Netherlands). Firstly, the ONT reads were assembled into contigs with the Flye assembler (v.2.5, Kolmogorov *et al.* 2019) using the following parameters: –nano-raw –estimated_genome_size 1400M –iterations 2. Contigs containing redundant sequences were removed with purge_haplotigs (commit 981bee4) using the following parameters: -l 15 -m 50 -h 70. Next, 2 rounds of polishing were performed with Illumina paired-end reads using pilon (v.1.23, Walker *et al.* 2014) which resulted in a final set of 296 contigs.

### Gene model prediction and functional annotation

RNA-seq based gene model prediction was performed using 8 RNA-seq samples (accession number PRJEB40906) including samples from young leaves, old leaves, and adventitious roots exposed to high light and low light conditions (Stander *et al.* 2020). The RNA-seq reads were individually aligned to the *V. minor* genome using HiSAT2 (v.2.2.1, Kim *et al.* 2019) and the resulting RNA-seq alignments were assembled into transcripts using StringTie (v.2.1.7, Pertea *et al.* 2015). Next, the 8 individual transcriptomes were merged using stringtie –merge into a non-redundant set of representative transcripts (v2.1.7, Pertea *et al.* 2015). The consensus transcriptome was then functionally annotated with the Trinotate pipeline (v3.0.1, Bryant *et al.* 2017) that combines BlastX (v.2.6.0-1, Camacho *et al.* 2009) and BlastP (v.2.6.0-1, Camacho *et al.* 2009) results from TransDecoder (v.5.5.0, Haas *et al.* 2013) predicted ORFs against the Uniprot database, and hmmscan (v.3.1b2, Finn *et al.* 2011) against the PFAM database (https://pfam.xfam.org/).

### Assembly completeness assessment

Assembly statistics were obtained using the stat bash program of the BBMap tool (v.38.94, Bushnell 2014). Genome assembly quality was also evaluate using the k-mer based method merqury (v.1.3, Rhie *et al.* 2020). Therefore, 20-mers database was constructed from Illumina short-reads using count function from meryl (v.1.3, Koren *et al*. 2018). Base level consensus quality score (QV) was estimated from k-mer survival rate. Genome coverage by Illumina short-reads was estimated to gain insight into the level of continuity of the final assembly. Reads were firstly aligned using bwa-mem2 (v.2.2.1, Vasimuddin *et al.* 2019). Resulting SAM file was sorted using samtools *sort* (v.1.16.1, Li *et al.* 2009) and the contig median coverage was calculated from samtools *coverage*. To assess the genome and gene annotation completeness, Benchmarking Universal Single-Copy Orthologs (BUSCO v.5.2.2, Simão *et al.* 2015) was applied with default setting using a plant-specific database of 2,326 single-copy orthologs. Number of annotated genes and associated statistics were obtained using agat_sp_statistics perl script from AGAT package (v.0.8.0, Dainat *et al.* 2022). Illumina RNA-seq reads used to perform gene annotation were mapped against the annotated genome using the splice-aware aligner STAR (v. 2.7.10a, Dobin *et al.* 2013) to gain more insight into the gene annotation quality.

### Transposable elements prediction and annotation

De novo transposable element (TE) identification and annotation were performed using the Extensive de novo TE Annotator pipeline (EDTA v.1.9.5, Ou *et al.* 2019) providing CDS sequences, enabling the identification of remaining TE (sensitive) using RepeatModeler (v.2.0.1, Flynn *et al*. 2020) and evaluating the classification consistency (evaluate). Long-terminal repeat (LTR) are annotated using LTR_finder (v. 1.07, Xu and Wang 2007) and LTRharvest included in GenomeTools (v.1.5.10, Ellinghaus *et al.* 2008). Terminal inverted repeat (TIR) are annotated using Generic repeat finder (v.1.0, Shi and Liang 2019) and TIR-learner (v.2.5, Su *et al.* 2019). *Helitrons* are annotated using HelitronScanner (v.1.1, Xiong *et al.* 2014).TIR candidates of less than 80 bp as well as LTR and *Helitrons* candidates of less than 100 bp are considered as tanderm repets and short sequences. False LTR discoveries are further filtered using LTR_retriever (v.2.9.0, Ou and Jiang 2018). Reported TIR candidates shorter than 600 bp are classified as MITEs. To remove false positives TIR and *Helitrons* candidates, several advanced filters are included in the EDTA pipeline (full-length copy numbers, simple sequence repeat, see Ou *et al.* 2019 for details). The obtained TE library is used to mask the genome and the remaining unmasked portion is scanned by RepeatModeler (v.2.0.1, default parameters, Flynn *et al*. 2020) to identify non-LTR retrotransposons and unclassified TE missed by structure-based TE identification tools. Finally, gene-related sequences have been removed using provided CDS.

Transposable enrichment in biosynthetic gene clusters (BGCs) was performed by comparing TEs proportion in BGCs to their proportion in the corresponding contig by an exact Poisson test with the poisson.test function implemented in stat package (v. 4.1.1) in R (v. 4.1.1, R Core Team 2020).

### GO enrichment

GO term enrichment analysis based on the GO classification was performed by comparing the relative occurrence of a GO term into increased orthogroups gene list to its relative occurrence in the genome by a Fisher’s Exact test (2-sided) with the R function topGO (v.2.44.0, Alexa and Rahnenfuhrer 2022). A Benjamini–Hochberg adjusted *P*-value of 0.05 was used as the cut-off criterion. Enriched GO terms were grouped based on semantic similarities using rrvgo package (Rel method, cut-off 0.7, v.1.4.0, Sayols 2020). Enriched terms graphs were performed using ggplot2 (v.3.3.5, Wickham 2011).

### Whole-genome duplication analysis

The DupPipe pipeline (Barker *et al.* 2010) was used to infer WGD events in *V. minor*, *Arabidopsis thaliana* (Lamesch *et al.* 2012), *C. roseus* (Franke *et al.* 2019), *Mitragyna speciosa* (Brose *et al.* 2021), *Solanum lycopersicum* (Hosmani *et al.* 2019), *Camptotheca acuminata* (Kang *et al.* 2021), *Calotropis gigantea* (Hoopes *et al.* 2018), *Gelsemium sempervirens* (Franke *et al.* 2019), *Ophiorrhiza pumila* (Rai *et al.* 2021), and *Papaver somniferum* (Guo *et al.* 2018). For each dataset, duplicated gene pairs were identified using a discontiguous MegaBLAST (Ma *et al.* 2002; Zhang *et al.* 2004) which selected sequences that shared at least 40% sequence similarity over 300 bp. The open reading frame for each gene pair was established by comparison to the NCBI’s plant RefSeqs protein database (2021 May 21) using BLASTX (v.2.6.0-1, Camacho *et al.* 2009) and retaining only the best hits with a sequence similarity threshold of 30% over 150 sites. GeneWise (Birney *et al.* 2004) was then used to align each gene DNA sequence against its best hit homologous protein sequence from the database in order to annotate its open reading frame and predict the resulting amino acid sequences for each gene. MUSCLE (v.3.6, Edgar 2004) then align the amino acids sequences for each gene pair to guide the nucleic acid alignments using RevTrans (v.1.4, Wernersson and Pedersen 2003). Finally, substitutions per synonymous site (Ks) are calculated using codeml’s F3x4 model from the PAML package (v.4.9, Yang 1997) to determine the divergence times between gene pairs. The resulting age distributions (Ks) were plotted using ggplot2 (v.3.3.3, Wickham 2011) in R (v.3.6.3, R Core Team 2013).

### Orthology analysis and phylogenetic tree reconstruction

Gene families were constructed by comparing the protein sequences of *V. minor* with 9 other plant species: *M. speciosa* (Brose *et al.* 2021), *G. sempervirens* (Franke *et al.* 2019), *C. roseus* (Franke *et al.* 2019), *C. gigantea* (Hoopes *et al.* 2018), *C. acuminata* (Kang *et al.* 2021), *O. pumila* (Rai *et al.* 2021), *S. lycopersicum* (Hosmani *et al.* 2019), *A. thaliana* (Lamesch *et al.* 2012), and *P. somniferum* (Guo *et al.* 2018). For each species, protein sequences with a length of < 30 amino acids were filtered out and protein sequences were clustered using CD-HIT (v.4.7, Fu *et al.* 2012) to select the longest representative sequence for each cluster. These representative sequences were then used as input for OrthoFinder (v.2.5.4, Emms and Kelly 2019) using the following parameters: -S diamond -t 100 -M msa -A muscle -T raxml-ng. A maximum-likelihood phylogenetic tree was obtained from OrthoFinder using the 799 single-copy orthogroups. Orthogroup gain and expansion were determined across the phylogenetic tree using Cafe5 (v.4.2.1, Mendes *et al.* 2020).

### Genome-wide synteny analysis

A genome-wide synteny analysis between *V. minor* genome and the genome of its closely relative species *C. roseus* (Franke *et al.* 2019) has been performed. Both genomes were aligned using minimap2 (v.2.24, Li 2018) using the following options: -cx asm20 –cs. The resulting paf file was visualized using D-Genies (https://dgenies.toulouse.inra.fr/, Cabanettes and Klopp 2018) selecting hits with at least 80% identity and sorting contigs by size.

### Identification of co-localized MIA gene regions on the *Vinca minor* genome and synteny analysis

Regions of co-localized biosynthetic genes were annotated on the *V. minor* genome using a personalized script that is available from https://doi.org/10.6084/m9.figshare.20749096.v1. Briefly, the script uses the genome .gff file, MIA blastn results in output format 6, and uniprot search results to anchor the putative MIA orthologs from the blastn analysis onto the genome. The script then searches 100,000 bp regions around each side of the annotation for additional genes annotated by pfam accessions: PF03171 (2OG-Fe(II) oxygenase superfamily), PF14226 (non-haem dioxygenase in morphine synthesis N-terminal), PF00891 (*O*-methyltransferase domain), PF08240 (Alcohol dehydrogenase GroES-like domain), PF00067 (Cytochrome P450), PF08031 (Berberine and berberine-like), and PF00201 (UDP-glucosyl transferase). Regions with more than one gene of interest are recorded as a cluster of interest.

Syntenic regions between the P450/OMT gene cluster for *V. minor* (scaffold 2420), *G. sempervirens* (scaffold 505, Franke *et al.* 2019), and *C. roseus* (scaffold 16, Franke *et al.* 2019) were compared using BLASTN (v.2.6.0-1, Camacho *et al.* 2009) with the following parameters: blastn -outfmt 6 -task blastn -perc_identity 70 -evalue 1e-10. The resulting hits between the clusters were filtered to only include alignments with an *E*-value of 1E-6 and alignment length of 700 bp and alignments were visualized using the R genoPlotR library (v0.8.11, Guy *et al.* 2010).

### Relative expression analysis

RNA-seq reads from young leaves (YL), old leaves (OL), and adventitious roots (AR) (Stander *et al.* 2020; RJEB40906) were pseudo-aligned onto the annotated transcripts from the *V. minor* genome using Salmon (v.0.14.1, Patro *et al.* 2017) with bias correction (-biasCorrect). Abundance estimates were established as transcripts per million (TPM) using the variational Bayesian optimized (-vbo) mode of Salmon.

### Template DNA construction for functional validation of candidate genes

*Vinca minor* MSTRG.7518 candidate was selected for functional validation based on its high nucleotide sequence identity with *C. roseus* T16Hs, co-expression with the validated Vm16OMT, and the synteny of this gene cluster with *C. roseus* and *G. sempervirens* methoxylation clusters. Given nucleotide sequence identity with T16H2, UTR-discriminating forward primer specific to *V. minor* MSTRG.7518 was designed to perform a 2-step PCR. In this respect, *V. minor* MSTRG.7518 was first amplified from *V. minor* cDNA using UTR forward primer and a reverse cloning primer (2 rounds), followed by the second PCR amplification of the obtained column-purified PCR product using cloning primers to introduce SpeI restriction sites (Supplementary Table 1). *Vinca minor* MSTRG.7518 was further ligated into donor DNA vector pPETA104 under pTEF1 promoter (Kulagina *et al.* 2021).

### Yeast strains

*Saccharomyces cerevisiae* CEN.PK113-7D (*MATa MAL2*-*8C, SUC2*) strain was transformed with Cas9-expressing pCfB2312 plasmid (Jessop‐Fabre *et al.* 2016) for downstream CRISPR/Cas9-assisted gene integration via lithium acetate transformation method (Chen *et al.* 1992). NotI-linearized template DNA was further co-transformed with gRNA helper plasmids pCfB3042 for the integration into X-4 locus (Mikkelsen *et al.* 2012) while *C. roseus* CPR2 was previously integrated into X-3 locus using pCfB3041. The transformants were selected on YPD agar plates (20 g l^−1^ peptone, 10 g l^−1^ yeast extract, 20 g l^−1^ glucose, 200 mg l^−1^ G418, and 100 mg l^−1^ nourseothricin) and screened by colony PCR for the integration of expression cassette (Supplementary Table 1).

### Culture conditions

Strain small-scale feedings were performed in 200 µl of YPD liquid media (20 g l^−1^ peptone, 10 g l^−1^ yeast extract, 20 g l^−1^ glucose) from the overnight 5 ml YPD cultures diluted 20 times (Kulagina *et al.* 2021), supplemented with 125 µM vincadifformine at 0 h, and glucose to 20 g l^−1^ at 24 h, during 48 h at 28°C and 200 rpm. The feedings were set up individually for each replicate and time point.

### Sample analysis

The supernatants were collected by centrifugation (10 min, 7,000 g) and diluted 20 times in MeOH 100%, followed by vortexing and 15 min centrifugation at 20,000 g prior to injection. Compound identification was performed by ultra-performance liquid chromatography-mass spectrometry (UPLC-MS) as described previously (Parage *et al.* 2016) with an 8-min linear gradient from 15 to 50% acetonitrile (containing 0.1% formic acid). The selected mode of ion monitoring was employed for the following compounds: vincadifformine (*m/z* 339), RT = 5.68; 16-hydroxyvincadifformine (*m/z* 355), RT = 4.22.

### Metabolomic analysis

Leaves, stems, flowers, and roots of *V. minor* were frozen in liquid nitrogen, then freeze-dried, ground into powder, and 2 replicates for each organ were sonicated with methanol and 0.1% of formic acid to extract metabolites. After centrifugations, extracts have been injected at 0.01 mg/ml on a UPLC system coupled to a quadrupole time-of-flight mass spectrometer, and the acquisition was made using full MS survey scans to acquire high-resolution MS1 data for metabolomics analysis and using a fast data-dependent acquisition to acquire MS/MS scans to help compounds identifications. Metabolomics data have been processed with MzMine2 (Pluskal *et al.* 2010). Peak areas have been corrected by dividing the peak area by the ratio: dry weight of plant/extract weight obtained in order to compare metabolite abundance between organs. More details are provided in Supplementary Information.

## Results and discussion

### *Vinca minor* genome sequencing, assembly, and analysis

**Fig. 2.**
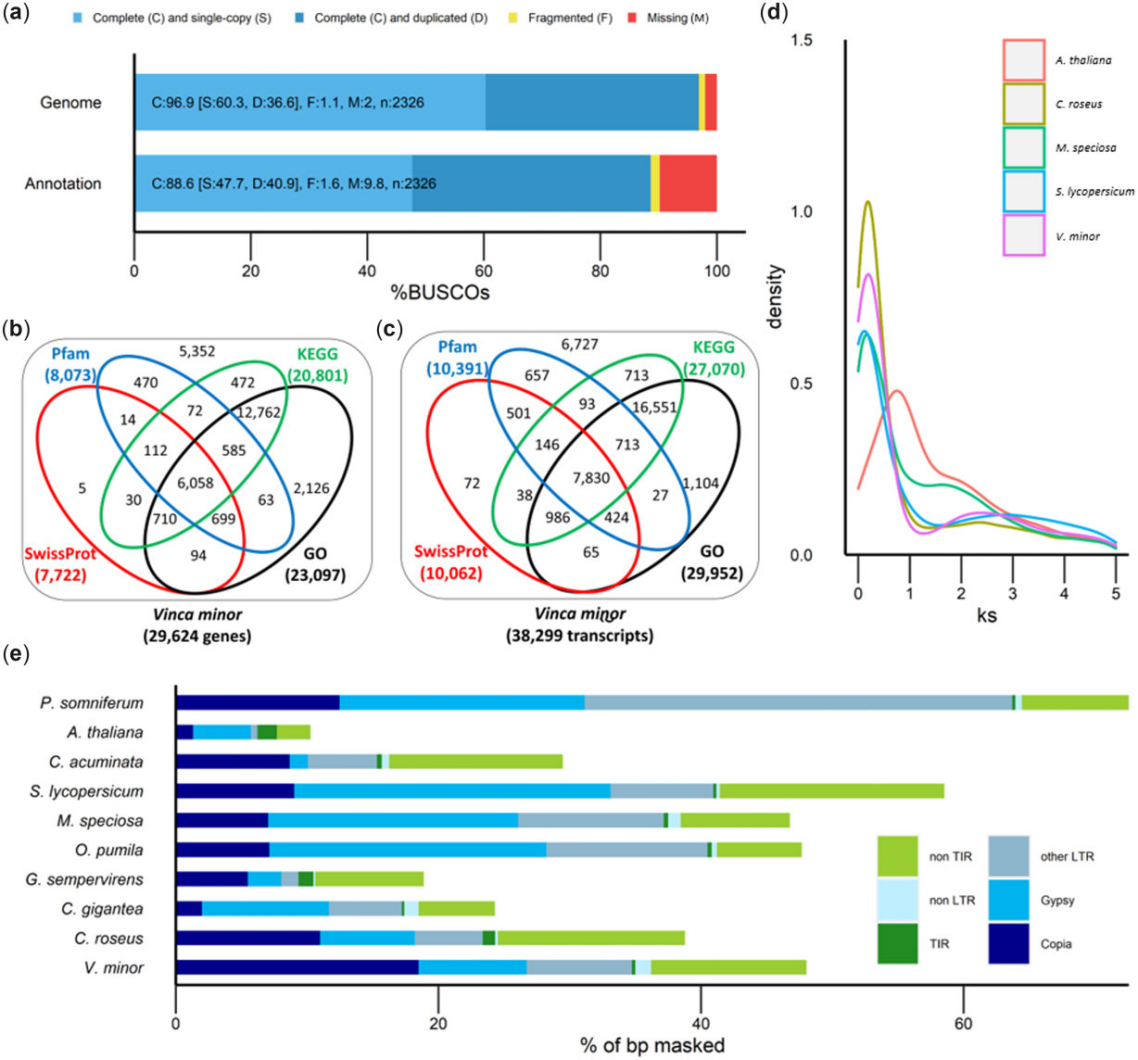
The annotated *Vinca minor* genome. a) BUSCO analysis of genome and annotated genes. b) Functional annotation of genes using SwissProt, Pfam, KEGG, and GO databases. c) Functional annotation of transcripts using SwissProt, Pfam, KEGG, and GO databases. d) Synonymous substitution rate (Ks) distribution plot for *V. minor* orthologs compared to other eudicots. e) TE proportion and classification. TE prediction and annotation have been performed using EDTA pipeline combining 6 prediction tools and one annotation step. TIR, terminal inverted repeat; LTR, long terminal repeat; other LTR, LTR containing retrotransposons except for Gypsy and Copia; non-LTR, retrotransposons without LTR sequence; non-TIR, DNA transposons without TIR sequence.

The *V. minor* genome was sequenced using ONT long-read sequencing, resulting in 6,006,337 reads representing 45.47 GB of data, as well as with Illumina short-read sequencing resulting in 177,102,894 reads representing 26.7 Gb of data (Supplementary Table 2). Illumina short-read k-mer counting estimated the *V. minor* genome length to be around 504,085 Mb, with the binomial distribution of the resulting k-mer graph typical of a diploid heterozygous genome with a heterozygosity rate of 1.54% (Table 2 and Supplementary Fig. 1). The genome was assembled from ONT long-reads using Flye (Kolmogorov *et al.* 2019). This Flye assembly consisted in 1,557.17 Mb distributed across 7,019 contigs with an N50 length of 0.5 Mb while its collapsing reduced length to 685.0 Mb, contig number to 296 and increased N50 length to 6.0 Mb. Finally, the collapsed assembly was polished twice using Illumina short-reads (Supplementary Table 2) with pilon (Walker *et al.* 2014), resulting in a final assembly length of 679 Mb (1.3 × the estimated assembly length), consisting of 296 scaffolds with an N50 scaffold length of 6.0 Mb (Table 2). 95.4% of this final assembly could be covered by Illumina short reads, indicating a good level of continuity. The base level QV of 28.6878 (Table 2), corresponding to more than 99.8% accuracy, highlights the good quality of this final assembly. Genome completeness was determined by identifying conserved orthologs from the eudicot lineage dataset in the genome using BUSCO (Benchmarking Universal Single-Copy Orthologs). This analysis revealed that 96.6% of 2,326 eudicot lineage genes could be identified in the genome, indicating a very high level of completion (Fig. 2a).

**Table 2.** *Vinca minor* genome assembly metrics.

Genome	*Vinca minor*
Estimated genome length (Mb)	504,085
Estimated heterozygosity (%)	1.54
Assembled genome length (Mb)	679,098
GC_content (%)	35.33
Total scaffolds	296
Scaffold N50 (Mb)	5.973
Scaffold N90 (Mb)	1.325
QV	28.6878
Predicted protein coding genes	29,624
Average gene length (bp)	5.016
Average transcripts per gene	1.3
Average CDS length (bp)	1186
Average exon per gene	6.6
Average intron length (bp)	661

RNA-seq-based gene model prediction using RNA-seq reads from the young leaves, old leaves, and adventitious roots of *V. minor* (Stander *et al.* 2020), were used to annotate a total of 29,624 protein-coding genes and 38,299 transcripts in the *V. minor* genome (Table 1), in comparison to the 34,363 and 22,617 transcripts reported in *C. roseus* and *G. sempervirens* (Franke *et al.* 2019). Depending on the RNAseq sample, 91.95–94.8% of reads uniquely mapped on this final assemble; 3.63–5.54% highlighted multiple matches and 1.19–2.51% did not match (Supplementary Table 3), indicating a good annotation quality. Among these 38,299 transcripts, 87.1% (33,357) presented a complete CDS based on TransDecoder ORF prediction. BUSCO analysis of the predicted transcripts revealed a complete BUSCO score of 88.6% and a low fragmentation score of 1.6%, indicating that a large number of genes with high-quality sequences have been annotated (Fig. 2a). These sequences were functionally annotated against Pfam, SwissProt, GO, and KEGG databases resulting in the annotation of 81.9% genes (24,272 predicted genes, Fig. 2b), and 82.4% transcripts (31,572 predicted transcripts, Supplementary Table 4; Fig. 2c). A quarter of the total genes were annotated by all 4 databases, whereas 53% of the genes were only annotated with GO and KEGG databases.

Next, we explored evolutionary WGD artifacts by identifying paralogous gene pairs across different plant species, and calculating the synonymous substitutions per synonymous site (Ks) for each gene pair (Fig. 2d). Sorting the paralogs in order of age (Ks) resulted in an initial high-density peak at low Ks representing the genes that are being duplicated at present. Over time, duplicated genes are lost which results in a L-shaped pattern or exponential decrease. Large-scale duplications, including WGD events, lead to substantial increases in the number of paralogs at a specific time point (Ks) which can be visualized as secondary peaks in the plot. The well-described and conserved γ whole-genome triplication event that is shared among eudicots (Jiao *et al.* 2012) was detected as such a secondary peak at around Ks = 2 in *V. minor*, *C. roseus*, *S. lycopersicum, O. pumila, C. gigantea, M. speciosa*, and *G. sempervirens* (Fig. 2d and Supplementary Fig. 2). *Vinca minor* did not show any additional secondary peaks in the Ks plot, suggesting a lack of any additional recent WGDs. However, the previously reported post-γ WGD (Kang *et al.* 2021; Rai *et al.* 2021) was evident in the paralog Ks plot of *C. acuminata* (Supplementary Fig. 2).

Lastly, we analyzed the composition in TEs of the *V. minor* genome. Repeat analysis showed that 48.03% of this genome consists of TEs, most of which being LTRs (34.74%) (Fig. 2e and Supplementary Table 5). Interestingly, the *V. minor* genome has the highest percentage of repetitive elements, compared to fellow *Gentianales* members, *C. roseus* (38.78%), *C. gigantea* (24.31%), *G. sempervirens* (19.38%), *O. pumila* (47.68%), and *M. speciosa* (46.76%, Fig. 2e). *Vinca* genome also present a higher TE proportion than *C. acuminata* (29.47%) and *A. thaliana* (10.26%) while *S. lycopersicum* (58.54%) and *P. somniferum* (72.58%) present a higher TE proportion than *V. minor*. Most LTRs were *Copia* elements in *V. minor* (18.5%), *G. sempervirens* (5.47%), *C. roseus* (11%), and *C. acuminata* (8.67%), whereas the 2 *Rubiaceae* species, *O. pumila* and *M. speciosa*, had a majority of *Gypsy* elements accounting for 21.09% and 19.05% of TEs in each genome, respectively (Fig. 2e and Supplementary Table 5). Such a distribution thus confirms that repeat elements are constantly expanding and contracting across different plant genomes, resulting in the disparity in genomic repeat profiles, even between closely related species (Lisch 2013).

### Comparative genomic analysis

**Fig. 3.**
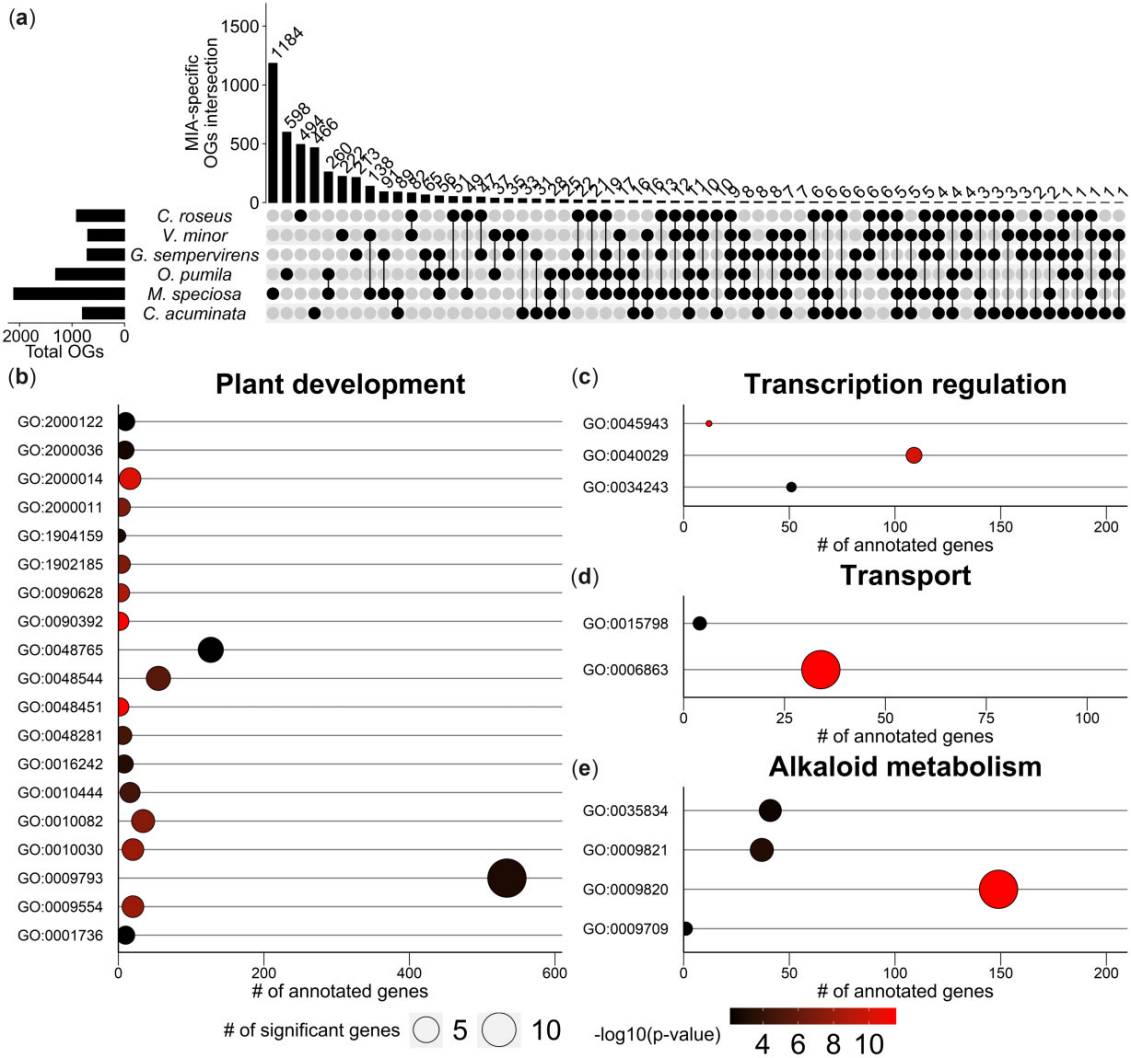
Comparative genomic analysis of *V. minor* with 9 additional plant species. a) The 4,592 orthogroups that are specific to the MIA-producing species; *V. minor*, *C. acuminata*, *C. roseus*, *G. sempervirens*, *M. speciosa*, and *O. pumila*. Numbers above bars correspond to the number of orthogroups present in each species which is represented by black-filled circles below the bars. The total number of orthogroups in each species is represented by the bars on the left axis. b–e) Enriched biological processes associated with plant development (19 GO terms, b), alkaloid metabolism (4 GO terms, c), transcription regulation (3 GO terms, d), and transport (2 GO terms, e). Number of genes in each GO term can be read on the x-axis. Circle size represents the number of significant genes in each GO term while its fill color highlights GO term significance level (Fisher’s exact test).

To attain additional insights into *V. minor* genome evolution, we compared the *V. minor* protein coding genes to the protein coding genes from 9 plant genomes, including 5 MIA-producing genomes (*C. acuminata*, *C. roseus*, *G. sempervirens*, *M. speciosa*, and *O. pumila*). OrthoFinder was used to generate orthogroups (OG, gene families) across these broader plant lineages. In total, 92.2% of genes were assigned to 23,486 OGs across all species (Supplementary Table 6), with a mean OG size of 11.8 proteins. Comparing orthogroups between MIA-producing and non-producing plants may provide interesting insights into which gene families are specifically conserved within the MIA-producing species. A total of 19,764 OGs were shared among the 6 MIA-producing plant proteomes (Supplementary Table 7). Of these, 4,592 OGs were specific to MIA-producers, and 11 OGs were common to all 6 MIA-producing species (Fig. 3a). Such conserved gene sets that are associated with MIA-production can shed light on the evolution and diversification of MIA-metabolism. For example, Gene ontology (GO) enrichment analysis revealed 387 biological processes, 84 cellular components and 154 molecular function GO terms enriched in *V. minor* genes present in the 19,764 OGs shared among the 6 MIA-producing plants. Among them, 20 are associated with transcription regulation (Supplementary Fig. 3), 75 to stimuli response including ABA, oomycetes, iron starvation, water deprivation, light, and 80 to plant development (including leaf development, vegetative-reproductive transition, cell growth, cell and nuclei division, PCD, secondary shoot development, and secondary cell wall development, autophagy). The *V. minor* genes present in the increased 4,592 OGs MIA-specific OGs (Fig. 3a) were found to be mainly implicated in biological processes associated with plant development (including endosperm development, endocytosis, petal formation, and seed germination: GO:0048451, GO:0090392, GO:2000014, GO:0090628, GO:0009554, GO:0010030, GO:0010082, GO:1902185, GO:2000011, GO:0048544, GO:0048281, GO:0010444, GO:0016242, GO:0009793, GO:1904159, GO:2000036, GO:0048765, GO:0001736, GO:2000122, Fig. 3b), transcription regulation (GO:0045943, GO:0040029, GO:0034243, Fig. 3c), transport (GO:0006863, GO:0015798, Fig. 3d), and most interestingly alkaloid metabolism (GO:0009820, GO:0009821, GO:0035834, GO:0009709, Fig. 3e).

**Fig. 4.**
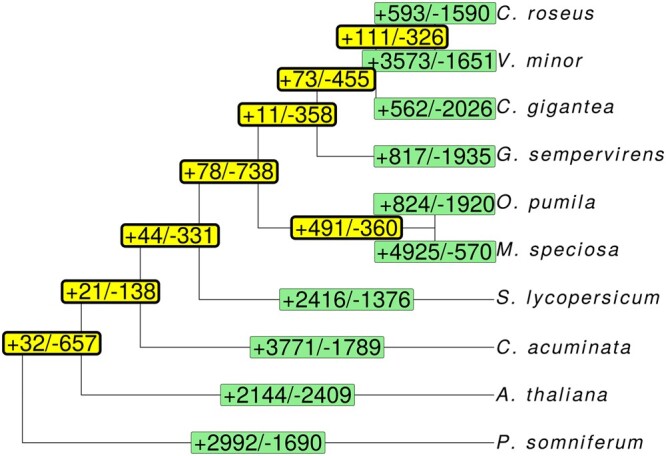
OrthoFinder phylogeny of *V. minor* with 9 other species. Changes in gene family sizes across the phylogenetic tree were calculated with Cafe5 (Mendes *et al.* 2020). Numbers in light bordered boxes correspond to gene families that were expanded (+) or contracted (−) in each lineage, whereas the numbers in the thick bordered boxes correspond to internal nodes of ancestral populations for each taxon.

To examine the phylogenetic relationships between the 9 investigated plant lineages, a maximum likelihood phylogenetic tree was derived by OrthoFinder from the 799 single-copy orthogroups (Supplementary Table 6) depicting the overt relationships among these species. Cafe5 (Mendes *et al.* 2020) was used to determine the lineage-specific (Fig. 4, green) and ancestral (Fig. 4, yellow) gene family evolution by investigating the expansion and contraction of orthogroups across the phylogenetic tree. Overall, the *V. minor* genome showed an increase and decrease of 3,573 and 1,651 OGs, respectively, which was the highest increase among the investigated *Apocynaceae* (Fig. 4). GO term enrichment analysis of expanded *V. minor* genes showed significant enrichment of biological processes associated with transcription regulation, response to stimuli (including ABA, oomycetes, iron starvation, water deprivation, and light) and plant development (including leaf development, vegetative-reproductive transition, cell growth, cell and nuclei division, PCD, secondary shoot development, secondary cell wall development, and autophagy) (Supplementary Fig. 3). A total of 823 orthogroups were determined to have undergone significant expansion/contraction (Supplementary Table 8, *P* < 0.05) of which 13 were associated with putative MIA pathway genes (Supplementary Table 9). These OGs included *V. minor* genes annotated for the upper pre-MIA pathway (*DL7GT*, *LAMT*), lower MIA pathway (*T16H2*, *16OMT*, *V19H*, *HYS*, *Redox2*, *SAT*), transcription factors (*BIS2* and *ZCT3*), and MIA transporters (*NPF2.4-2.7*, *NPF2.9*, *MATE2*) (Pauw *et al.* 2004; Levac *et al.* 2008; Murata *et al.* 2008; Salim and De Luca 2013; Besseau *et al.* 2013; Stavrinides *et al.* 2016; Van Moerkercke *et al.* 2016; Larsen *et al.* 2017; Payne *et al.* 2017; Qu *et al.* 2019; Williams *et al.* 2019). Such evolutionary dynamics could explain the biochemical diversity displayed between closely related MIA-producing species, for example as seen between *C. roseus* and *V. minor.*

### *Vinca minor* metabolomics analysis and homology-based prediction of putative MIA gene orthologs

To guide future elucidations of MIA biogenesis in *V. minor*, we investigated the specific content of MIA biosynthetic genes of this plant in comparison to MIA content. A UPLC/HRMS analysis was thus performed on methanolic extracts of *V. minor* roots, stems, flowers, and leaves. Principal component analysis showed sample clustering per organ type thus highlighting the specificity of MIA accumulation in each organ with roots being the most different (Supplementary Fig. 4). Among the studied MIAs, 22 were identified at different confidence levels (Supplementary Table 10). As previously observed, vincamine was the most abundant MIA in the 4 organs with high amounts of its 9-methoxy derivative in leaves and stems (Supplementary Fig. 5) (Abouzeid *et al*. 2017). Interestingly, while vincadifformine was accumulated at low or trace levels in all organs, numerous derivatives were identified including minovincinine, 16-methoxyvincadifformine (ervinceine), 11-methoxyminovincinine (16-methoxyminovincinine), minovincine, and minovine for instance, especially in leaves and flowers. We also observed numerous 11/16-methoxy derivatives of vincadifformine including 16-methoxyvincadifformine (ervinceine), 11-methoxyminovincinine (16-methoxyminovincinine), and 11 methoxyminovine (16-methoxyminovine). By contrast, no acetylated derivatives of vincadifformine such as echitovenine were detected and neither tetrahydroalstonine, ajmalicine, tabersonine and derivatives (lochnericine, hörhammericine) or catharanthine as expected. At the gene level, since *C. roseus* and *Rauwolfia* species have been widely used to elucidate MIA routes, we used functionally validated MIA pathway genes of these plants in BLAST searches to identify the corresponding predicted *V. minor* orthologs (Supplementary Table 9). Orthologs of all genes from the methylerythritol and monoterpene secoiridoid pathways, which provide secologanin, were identified. We thus observed that *V. minor* displays 4 potential copies of secologanin synthase (MSTRG.3111, MSTRG.22185, MSTRG.1442, and MSTRG.1443) as observed previously in *C. roseus* (Kellner *et al.* 2015b). In addition, orthologs of strictosidine synthase (*STR*) and strictosidine b-glucosidase (*SGD*) that ensure strictosidine formation and deglycosylation for downstream MIA synthesis were predicted (McKnight *et al.* 1990; Gerasimenko *et al.* 2002). Potential orthologs of all genes ensuring vincadifformine synthesis from geissoschizine synthase (*GS*) up to VS were also retrieved from the BLAST analysis (Tatsis *et al.* 2017; Caputi *et al.* 2018; Qu *et al.* 2019). Besides the 2 potential copies of VS as described in *C. roseus*, we also predicted 4 orthologs of tabersonine synthase (*TS*) (Caputi *et al.* 2018; Qu *et al.* 2019). Since tabersonine is not accumulated in *V. minor*, it is highly possible that these genes catalyze the synthesis of other MIAs of interest. By contrast, we did not find any potential true orthologues of catharanthine synthase (*CS*) and minovincinine 19-hydroxy-*O*-acetyltransferase (*MAT*) in agreement with the non-detection of catharanthine and echitovenine in *V. minor*. Finally, already known genes from *V. minor* including *VmPiNMT*, *Vm16OMT*, and *VmTPT2* were also identified. In conclusion, all the results illustrated how a genome-based prediction of MIA biosynthetic genes can directly provide potential new gene candidates as exemplified by the *TS* orthologues. Furthermore, such a prediction could also be of high interest to guide the discovery of yet unknown MIA biosynthetic steps by mining the genomic environment of the predicted orthologues.

### Identification of MIA gene clusters in *Vinca minor* led to the identification of a functional vincadifformine 16-hydroxylase

It has been well described that genes involved in similar specialized metabolisms can cluster on genomic regions in fungi and bacteria as well as in plant genomes (Nützmann *et al.* 2018). The current definition for plant BGCs states that BGCs contain at least 3 different types of nonhomologous enzymes and are typically co-expressed (Nützmann *et al.* 2016). However, additional genes sharing high-homology to cluster genes may exist within these regions, having arisen from tandem duplications. We, therefore, extended our *V. minor* genetic investigation by searching for co-localization of metabolic genes of interest in the *V. minor* genome. The predicted MIA orthologs (Supplementary Table 9) were thus anchored onto the *V. minor* genome and used to search the surrounding genomic landscapes in order to identify associated biosynthetic genes or gene duplicates. On this basis, by using a custom built script, we captured the genomic regions that contained at least 2 genes of interest or gene duplicates, including alcohol dehydrogenases, dioxygenases, hydrolases, methyltransferases, cytochrome P450s, transcription factors, acetyltransferases, and UDP-glycosyltransferases, which constitute the enzyme arsenal mostly involved in specialized metabolisms. A total of 23 putative co-localized gene regions of interest were identified comprising 387 genes and 16 putative MIA biosynthetic genes of interest (Supplementary Table 11). For instance, in gene cluster 1, a total of 2 cytochrome P450s, 3 acetyltransferases, 1 hydrolase, 1 alcohol dehydrogenases, and 1 transcription factors clustered together in a region of 482 kb, some of them corresponding to putative orthologues of MIA biosynthetic genes. It is thus highly possible that this region corresponds to a potential reservoir of genes encoding yet unknown enzymes of the MIA pathway.

**Fig. 5.**
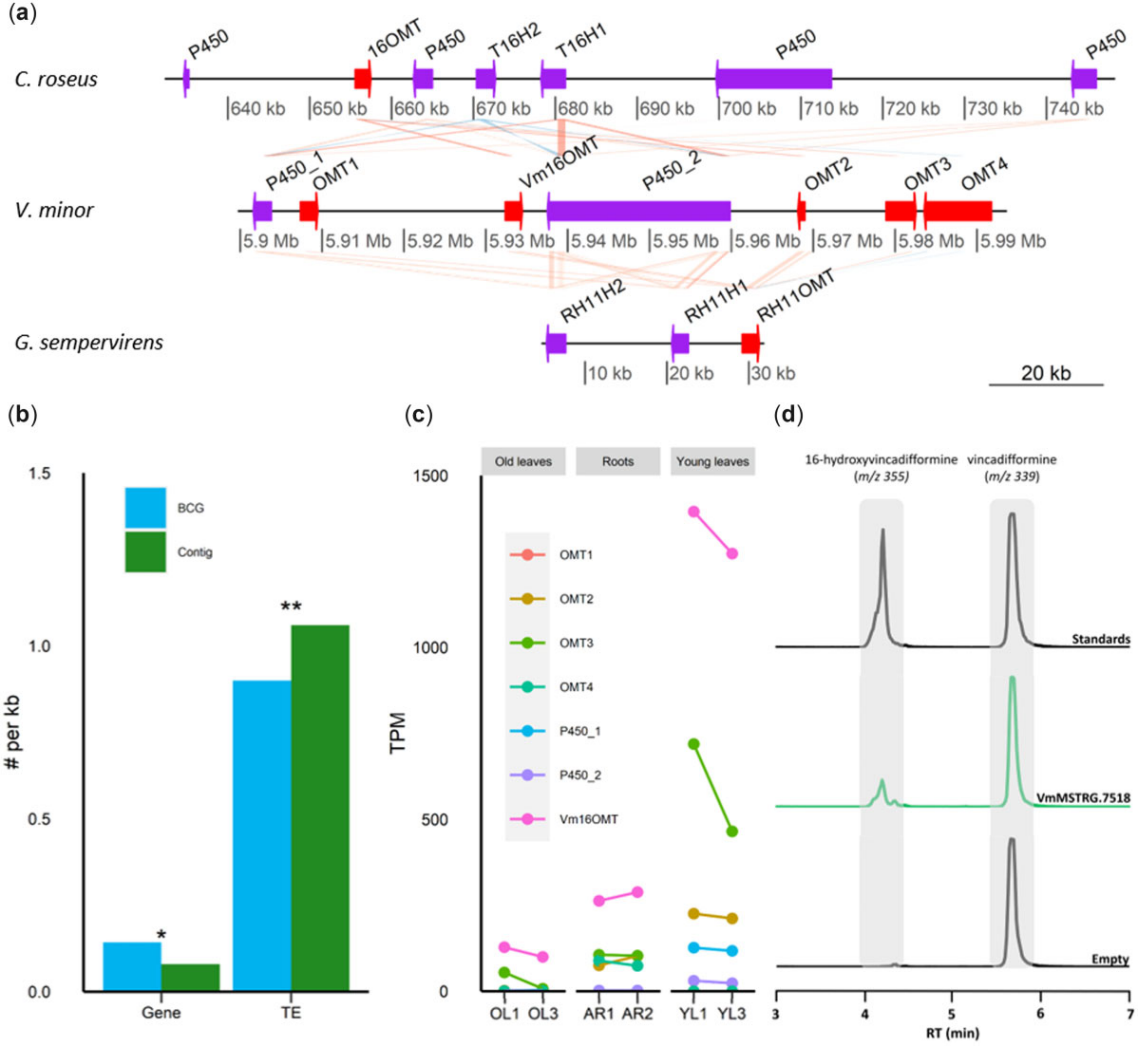
A syntenic *V. minor* OMT-cytochrome P450 gene cluster guides the identification of a functional vincadifformine hydroxylase. a) Synteny between *C. roseus*, *V. minor* and *G. sempervirens* scaffolds that are involved in the methoxylation of indoles. b) Gene and TE proportion in cluster 675 compared to scaffold 2420. *P*-value: 0 “***” 0.001 “**” 0.01 “*” 0.05 “NS” 1. c) Relative expression levels, quantified as TPM, for annotated hydroxylases (P450_1: MSTRG.7518 and P450_2: MSTRG.7522) and *O*-methyltransferases *(Vm16OMT* and *OMT1–OMT4*). d) VmMSTRG.7518-expressing yeast strains were fed with 125 µM of vincadifformine during 48 h along with the negative control strain (CrCPR2-expressing strain with integration-free X-4 chromosome site; referred to as “Empty”). Three technical replicates of the 3 biological replicates were further analyzed by UPLC-MS for VmMSTRG.7518-expressing yeast strains, and 3 technical replicates for the negative control strain. Selected ion monitoring method (vincadifformine: *m/z* 339; 16-hydroxyvincadifformine: *m/z* 355) along with the standards were employed to detect vincadifformine or the hydroxylated product of vincadifformine. The representative replicate results are displayed. RT, retention time.

In addition, physically co-localized genes may also be conserved between chromosomes within or between species which is known as synteny. A synteny analysis was thus performed between *V. minor* and the well-characterized *C. roseus*. The currently available *C. roseus* assembly (Franke *et al.* 2019) is still highly fragmented and contigs can be misoriented. The dot plot comparing *V. minor* and *C. roseus* clearly indicated an apparent collinearity between the 2 genomes albeit it can be partially masked due to improper contig orientation and fragmentation in *C. roseus* (Supplementary Fig. 6a). However, we were able to find a conserved organization with 237 of the 296 *V. minor* contigs having at least one hit with a *C. roseus* contig and 438 of the 2,090 *C. roseus* contigs having at least one hit with a *V. minor* contig (Supplementary Fig. 6, a and b and Table 12). Among the other contigs, 197 *V. minor* contigs and 394 *C. roseus* contigs display several matches (Supplementary Fig. 6, a and b) as exemplified with *C. roseus* contig 60 and *V. minor* contig 4699 (Supplementary Fig. 6c). Indeed, 1 sterol transport-associated gene (MSTRG.18143), 2 SCARECROW-like transcription factor (MSTRG.18145, MSTRG18147), 1 LACCASE (MSTRG.18149), and 1 metalloprotease (MSTRG.18150) can be found in the 70 kb-long fragment of *V minor* contig 4699. A similar gene content and organization can be observed in the 110 kb-long syntenic fragment of *C. roseus* contig 60, composed of 1 metalloprotease (CRO_133159), 1 LACCASE (CRO_133160), 4 SCARECROW-like (CRO_133163, CRO_133164, CRO_133165, CRO_133166), and 1 sterol transport-associated gene (CRO_133167). On the other hand, it is well known that synteny between different species may unveil related genes that are involved in specific metabolite biosynthesis and allow the transfer of gene discoveries made in one species to phylogenetically related species. For example, in *C. roseus*, 2 genes coding *T16H* isoforms (*T16H1* and *T16H2*) with the associated *16OMT* were found to be physically co-located on the *C. roseu*s genome (Franke *et al.* 2019; Kellner *et al.* 2015b). By microsynteny analysis, we identified a similar methoxylation gene cluster (cluster 22) in *V. minor* on contig_2420 (Fig. 5a and Supplementary Table 11). This cluster contained 2 putative cytochrome P450s (MSTRG.7518 and MSTRG.7523) with high homology to *C. roseus T16Hs* (78.4% and 77.4 nucleotide sequence identity, Supplementary Tables 9 and 11), as well as 5 methyltransferases (MSTRG.7519, MSTRG.7522, MSTRG.7526, MSTRG.7528, and MSTRG.7529; 63.5–75.6% nucleotide sequence identity to *C. roseus16OMT,*Supplementary Tables 9 and 11), including the functionally validated *Vm16OMT* (MSTRG.7522) that was found to methylate 16-hydroxyvincadifformine (Stander *et al.* 2020). Interestingly, this gene organization also corresponds to the methoxylation cluster identified in *G. sempervirens* composed of rankinidine and humantenine-11-hydroxylase (*RH11*) and rankinidine and humantenine-11-*O*-methyltransferase (*RH11OMT*) which catalyze the hydroxylation and methylation of the MIA oxindole scaffold in *G. sempervirens* (Franke *et al.* 2019). As expected, the synteny comparison between *V. minor* scaffold contig_2420, *C. roseus* scaffold cro_v2_scaffold_16 and *G. sempervirens* scaffold_505 thus revealed considerable similarities between the 3 methoxylation clusters. Furthermore, we compared the relative proportion of genes and TEs in cluster 22 to their proportion on scaffold 2420 (Fig. 5b). Eighteen genes and 176 TE have been annotated on cluster 2 whilst 510 genes and 6,780 TE have been annotated on scaffold 2420. An exact Poisson test revealed a significant gene enrichment (*P*-value = 0.004751285) in cluster 2 as compared to scaffold 2420. This result is in agreement with the already described TEs enrichment in numerous BGCs (Field *et al.* 2011; Field and Osbourn 2012; Winzer *et al.* 2012; Krokida *et al.* 2013; Li *et al.* 2021; Shen *et al.* 2021), supporting TEs involvement in the formation of metabolic gene clusters by providing homologous sequences for genomic recombinations and rearrangements (Huang *et al.* 2012; Bennetzen and Wang 2014).

To further describe the putative *V. minor* methoxylation cluster, we next calculated the relative expression level of each gene as TPM using data from the *V. minor* transcriptome (Stander *et al.* 2020; Fig. 5c). The highest expression levels were obtained for the *O*-methyltransferases *Vm16OMT,OMT3,* and *OMT2* in young leaves where MIA metabolism is high. While P450_2 was only expressed at trace levels in the studied organs, P450_1 was substantially expressed in young leaves (>100 TPM) which prompted us to perform its functional validation. To conduct this assay, P450_1 (VmMSTRG.7518) was expressed in yeast following a CRISPR/Cas9-mediated integration. The resulting yeast strain, as well as a strain transformed by an empty vector, were then cultured and fed with vincadifformine before analysis of the resulting products with ultra-performance liquid chromatography-mass spectrometry (UPLC–MS; Fig. 5d). The comparison of selected ions (*m*/*z* 339 for vincadifformine, *m*/*z* 355 for 16-hydroxyvincadifformine) to retention times of standards allowed to establish that P450_1 (VmMSTRG.7518) hydroxylated vincadifformine into 16-hydroxyvincadifformine as compared to yeast transformed with empty vector. This result thus confirms that VmMSTRG.7518 and *Vm16OMT* form a vincadifformine methoxylation cluster responsible for the formation of 16-methoxyvincadifformine (ervinceine) in leaves of *V. minor*. This throws light on the conservation of a methoxylation gene cluster organization in *V. minor*, *C. roseus*, and *G. sempervirens*.

## Conclusion

Here, we describe the genome of the lesser periwinkle, *V. minor*, which is the first *Apocynaceae* native to central and southern Europe sequenced to date (Fig. 1c). While *V. minor* shares numerous genomic and evolutionary traits with the other sequenced MIA producing plants, the careful examination of gene organization also sheds light on the existence of several putative gene clusters whose future functional characterization will probably lead to the discovery of new MIA biosynthetic enzymes. Similarly, gene synteny analysis combined with gene functional validation allowed the identification of *V. minor* vincadifformine methoxylation cluster as already described in *C. roseus* and *G. sempervirens* for tabersonine and rankinidine, respectively. This reinforces the potential interest of this genomic data set for gene discovery and contributes to the construction of an MIA-orientated pan-genome. Therefore, beyond widening our knowledge on MIA biosynthesis, the future elucidations of MIA biosynthetic routes resulting from this study will also allow the development of new MIA supply approaches based on the gene transfer in heterologous hosts such as yeast or bacterium (Courdavault *et al.* 2021). These metabolic engineering strategies allowed creating efficient cell factories producing natural products on demand to face unstable and limited production in plants, as reported for strictosidine, vindoline, and vinblastine (Brown *et al.* 2015; Kulagina *et al.* 2021; Liu *et al.* 2021; Zhang *et al.* 2022).

## Supplementary Material

jkac268_Supplemental_Materials_and_Methods_and_Figures

jkac268_Supplemental_Tables

## Data Availability

Raw DNA-seq and the genome assembly have been deposited in the NCBI database under the BioProject accession number: PRJNA873287 (https://www.ncbi.nlm.nih.gov/bioproject/PRJNA873287). The genome annotation, transcript expression abundances, and script used for searching for MIA gene clusters are available on the figshare: https://doi.org/10.6084/m9.figshare.20749096.v1. Supplemental material available at G3 online.
