# Supplementary material for: The Vinca minor genome highlights conserved evolutionary traits in monoterpene indole alkaloid synthesis
Source: G3 (Bethesda). 2022 Oct 6;12(12):jkac268. doi: 10.1093/g3journal/jkac268 (PMC9713385; doi:10.1093/g3journal/jkac268)
Supplement: jkac268_Supplemental_Materials_and_Methods_and_Figures [file jkac268_supplemental_materials_and_methods_and_figures.docx]

Supporting Information for “The *Vinca minor* genome highlights conserved evolutionary traits in monoterpene indole alkaloid synthesis”

By Emily Amor Stander et al.

**Table of contents**

1. **Supplemental Materials and Methods: Metabolomics analysis (p.1)**
2. **Supplemental Figures (p.3):**

**Figure S1.** Genome size and heterozygosity estimation using GenomeScope based on 21-mers from Illumina short-reads.

**Figure S2.** Ks (synonymous substitution rate) density plots depicting the distribution of paralogous gene pairs for selected available plant genomes.

**Figure S3.** Gene ontology (GO) enrichment analysis of expanded *V. minor* genes showing significant enrichment of genes associated with (A) transcription regulation , including stimuli response (including ABA, oomycetes, iron starvation, water deprivation, light, B) response to stimuli and C) plant development (including leaf development, vegetative-reproductive transition, cell growth, cell and nuclei division, PCD, secondary shoot development, secondary cell wall development, autophagy)

**Figure S4.** Clustering of samples into groups of biological replicates.

**Figure S5.** UPLC/HRMS peak area comparison of 22 alkaloids compounds identified in *Vinca minor* plant’s organs

**Figure S6.** Synteny between V. minor and C. roseus genomes. (A) Genome-wide synteny. (B) Number of contig from V. minor (blue) and C. roseus (red) with at least one hit. (C) Focus on a region of C. roseus contig 60 and V. minor contig 4699. Arrows highlight gene orientation. Green: transcription factor (SCARECROW-like), Red: LACCASE, Blue: Sterol transport associated gene, Brown: Metalloprotease, Black: hydrolase, Grey: other function, White: not annotated. Dark blue: antisens match.

1. **Results: Phytochemical investigation of *Vinca minor.* (p. 9)**
2. **References (p10)**

**Supplemental Materials and Methods: Metabolomics analysis.**

*Chemicals*

Methanol purchased from Fisher, HPLC gradient grade, was used for the extraction of the phytochemicals with formic acid from Fluka. Acetonitrile LC-MS grade solvent was purchased from Fisher, Optima LC/MS grade. Standards were purchased from Biosynthese Carbosynthese for Vincadifformine, from Extrasynthese for Vincamine, from Phytoconsult for Strictosidine, and nicely sent from our collaborators for Geissoschizine, Akuammicine, Pleiocarpamine and Picrinine. Ultra-pure water, obtained from a Direct-Q system (Millipore, Billerica, MA, USA) was used throughout the analysis.

*Plant materials*

Leaves, stems, flowers, and roots of *V. minor* were collected on the University of Tours campus during spring 2021 (Tours, France). The plant materials were frozen in liquid nitrogen, then freeze-dried using an Alpha 1-2 LD plus Christ freeze dryer (Christ, Osterode am Harz, Germany) and crushed into powder using an Ika A10 grinding machine (Ika, Staufen, Germany).

*Extraction*

Extractions were processed for two replicates of each plant organ, leaf, stem, root, and flower leading to 12 samples. The powdered plant materials (50 mg dry weight for each sample) were placed in a glass tube with 1 ml of methanol with 0.1 % formic acid and sonicated for 1h. Supernatants were separated from the residuals powder by 15 min centrifugation at 4000 G at 4°C, followed by a second identical centrifugation process before being evaporated in a speed-vac. Extracts were re-suspended in MeOH at 0.01 mg/mL prior to LC/MS analysis.

*HPLC/ESI-QTOF/MS conditions and MS data acquisition*

Analyses were carried out on a Waters Acquity UPLC system (Waters Co., Milford, MA, USA) coupled to a Waters Synapt G2-Si HDMS quadrupole time-of-flight mass spectrometer (Waters MS Technologies, Manchester, UK) equipped with an electrospray ionization interface (ESI). Chromatographic separations were performed on a Waters Acquity UPLC BEH C_18_ (100*2.1 mm, 1.7 µm) column, and the temperature was maintained at 40°C. Mixtures of H_2_O (A) and ACN (B) were eluted as flow rate of 0.4 mL/min with a gradient as follow: 10-60% B (0-12 min), 100% B (12-16 min), 10% B (16-20 min). The samples (1 µL injection volume) were analyzed in fast data-dependent acquisition (fDDA) mode consisting of a full MS survey scan in the *m/z* 50/1200 Da range (scan time = 0.2 ms) followed by MS/MS scans for the three most intense ions (*m/z* 100-1200 Da; scan time = 0.05 ms). The gradient of collision energy was set as 10-40 V for low-mass and 40-90 V for high-mass. The samples (1µL injection volume) were also analyzed in MS mode consisting of full MS survey scan in the *m/z* 100/1200 Da range to acquire high-resolution MS1 data.

*Processing of mass spectrometry data*

The accurate mass data of the [M+H] ions and the MS/MS product ions were processed using Mass Lynx software (v.4.2 SCN983; Waters). Files were transformed into mzXML format with MSConvert software, part of the ProteoWizard package (Chambers et al., 2012). All .mzXML were then processed using MzMine2 (Pluskal et al., 2010). The mass detection was realized keeping the noise level at 10^3. The ADAP chromatogram builder was used with a minimum group size of scans of 5, a group intensity threshold of 10^3, a minimum highest intensity of 10^3, and *m/z* tolerance of 0.01 Da. The ADAP wavelets deconvolution algorithm was used with the following settings: S/N threshold = 10, minimum feature height = 1000, coefficient/area threshold = 110, peak duration range 0.02 - 1 min, RT wavelet range 0.00 – 0.05. Isotopologues were grouped using the isotopic peaks grouper algorithm with an *m/z* tolerance of 0.001 Da and an RT tolerance of 0.1 min. Peak alignment was performed using the join aligner module (m/z tolerance = 0.01 Da , weight for m/z = 0.5, weight for RT = 0.5, absolute RT tolerance 0.1 min).

*Statistical analysis*

Peak area correction, sample injection controls, principal coordinate analysis (PCA), and boxplots were carried out in R (v 4.0.2) (R Core Team, 2021). In order to compare metabolites abundance between organs, peak areas were divided by the ratio dry weight of plant/ extract weight obtained for each sample. For sample injection control, PCA of samples and quality control (QC) was performed, and QCs were removed from the peak table after verifying that they cluster together and apart from other samples on the PCA plot (Lê et al., 2008).

**Supplemental Figures**

**
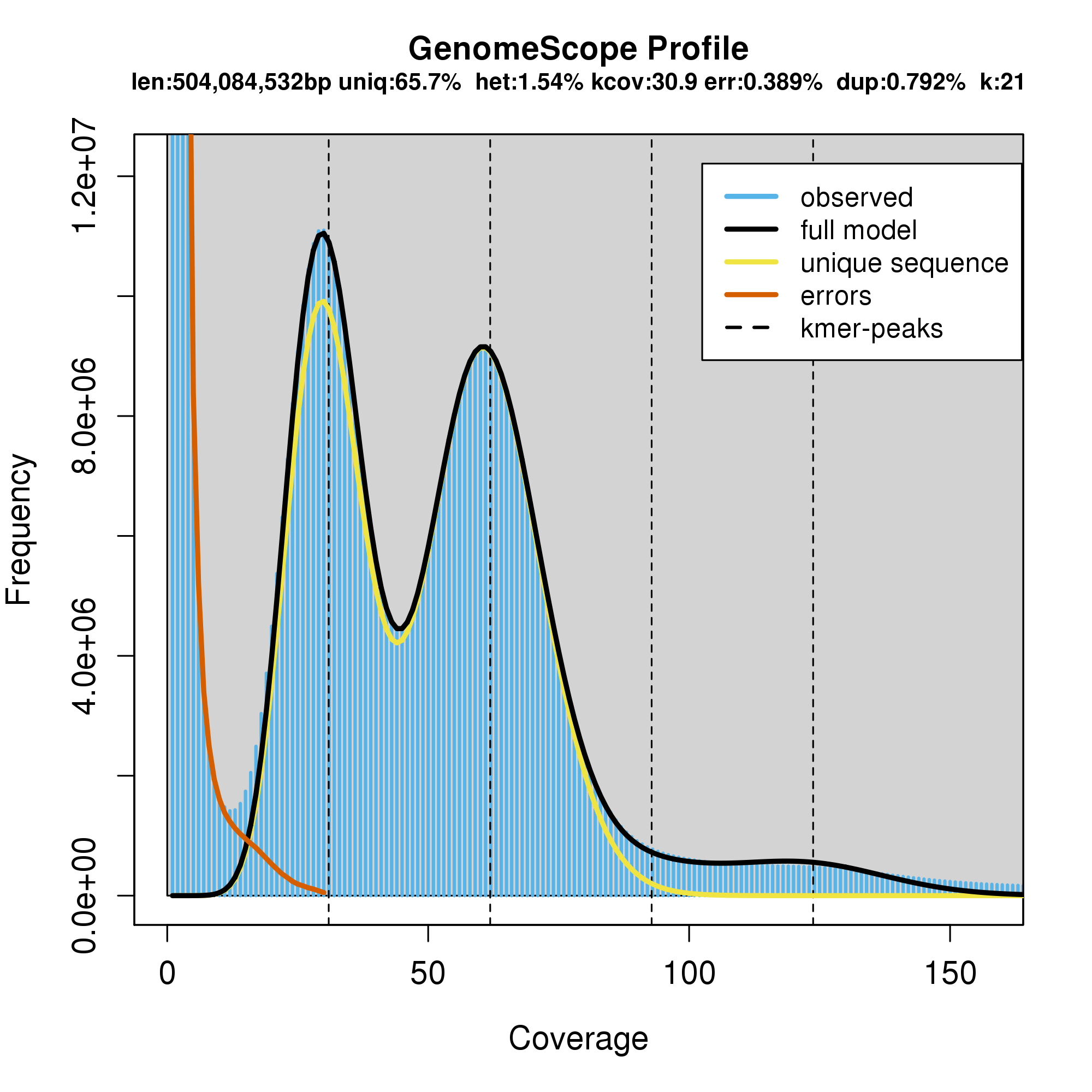
**

**Figure S1.** Genome size and heterozygosity estimation using GenomeScope based on 21-mers from Illumina short-reads.

**
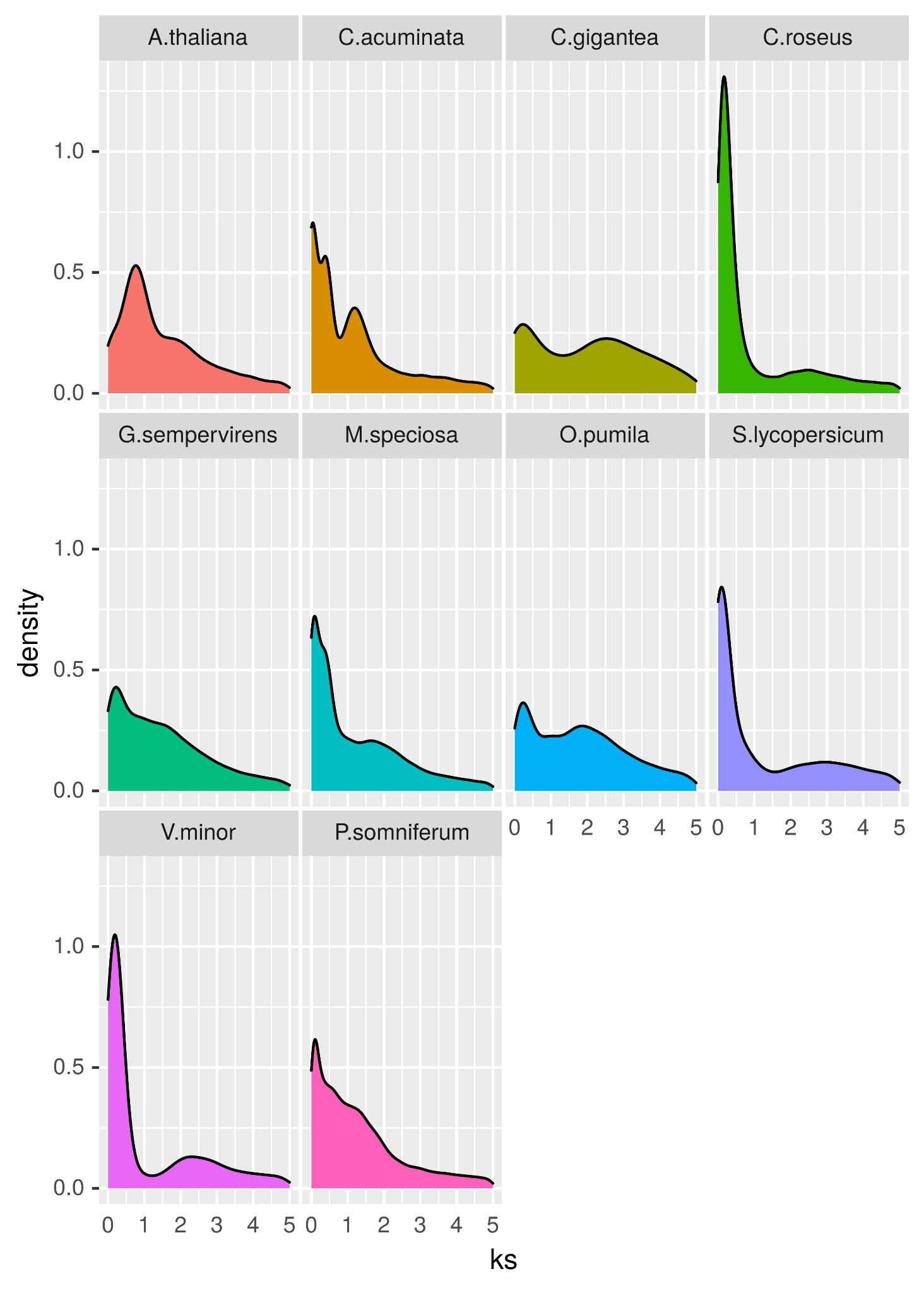
**

**Figure S2.** Ks (synonymous substitution rate) density plots depicting the distribution of paralogous gene pairs for selected available plant genomes.


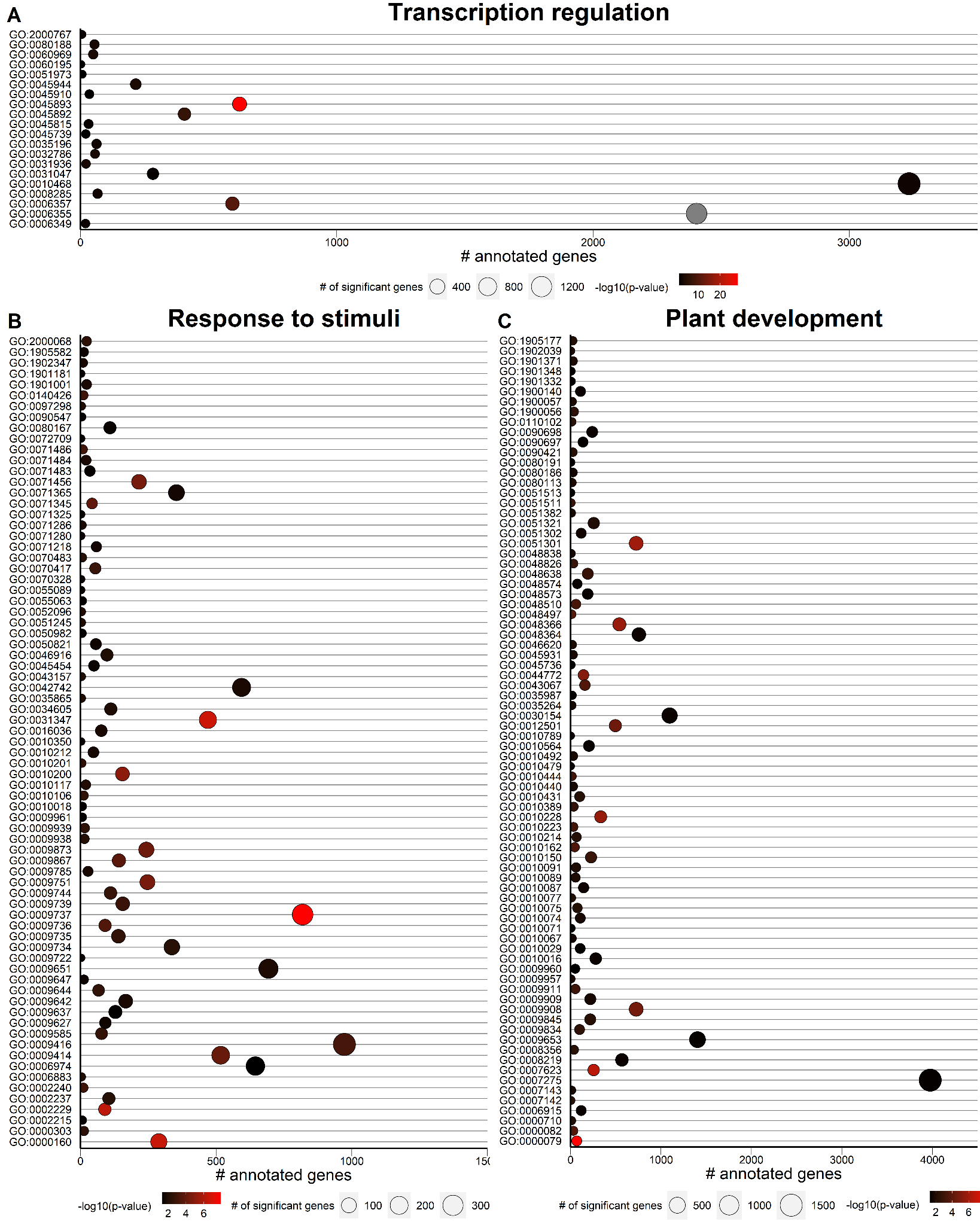


**Figure S3.** Gene ontology (GO) enrichment analysis of expanded *V. minor* genes showing significant enrichment of genes associated with (A) transcription regulation , including stimuli response (including ABA, oomycetes, iron starvation, water deprivation, light, B) response to stimuli and C) plant development (including leaf development, vegetative-reproductive transition, cell growth, cell and nuclei division, PCD, secondary shoot development, secondary cell wall development, autophagy)


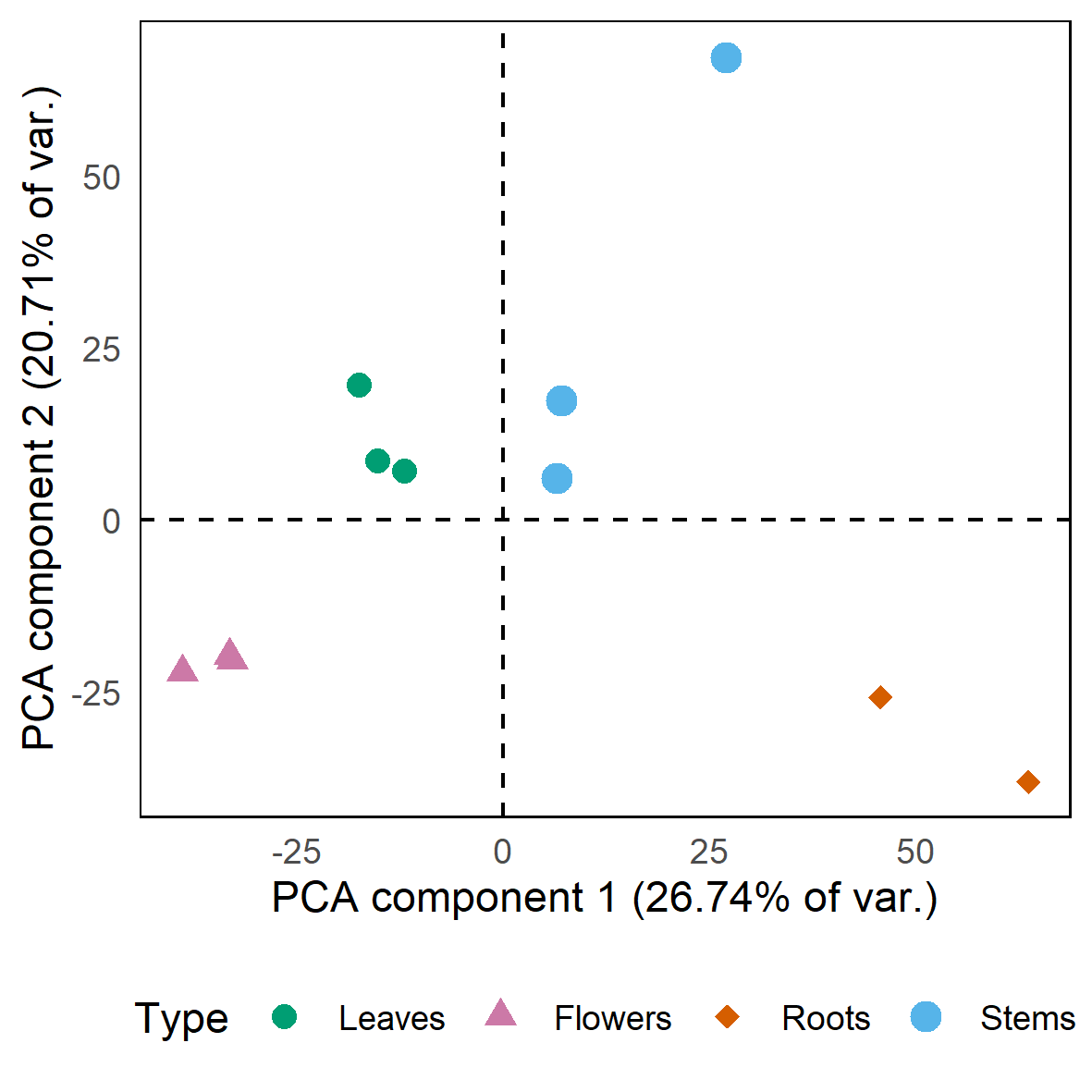


**Figure S4.** Clustering of samples into groups of biological replicates. Principal component analysis (PCA) of the peaks table of *Vinca minor*‘s samples: leaves, flowers, roots and stems extracts. The metabolome of *Vinca minor*’s roots is more specific compared to the metabolome of *Vinca minor*’s leaves, flowers, and stems samples which are showing more similarities between them.


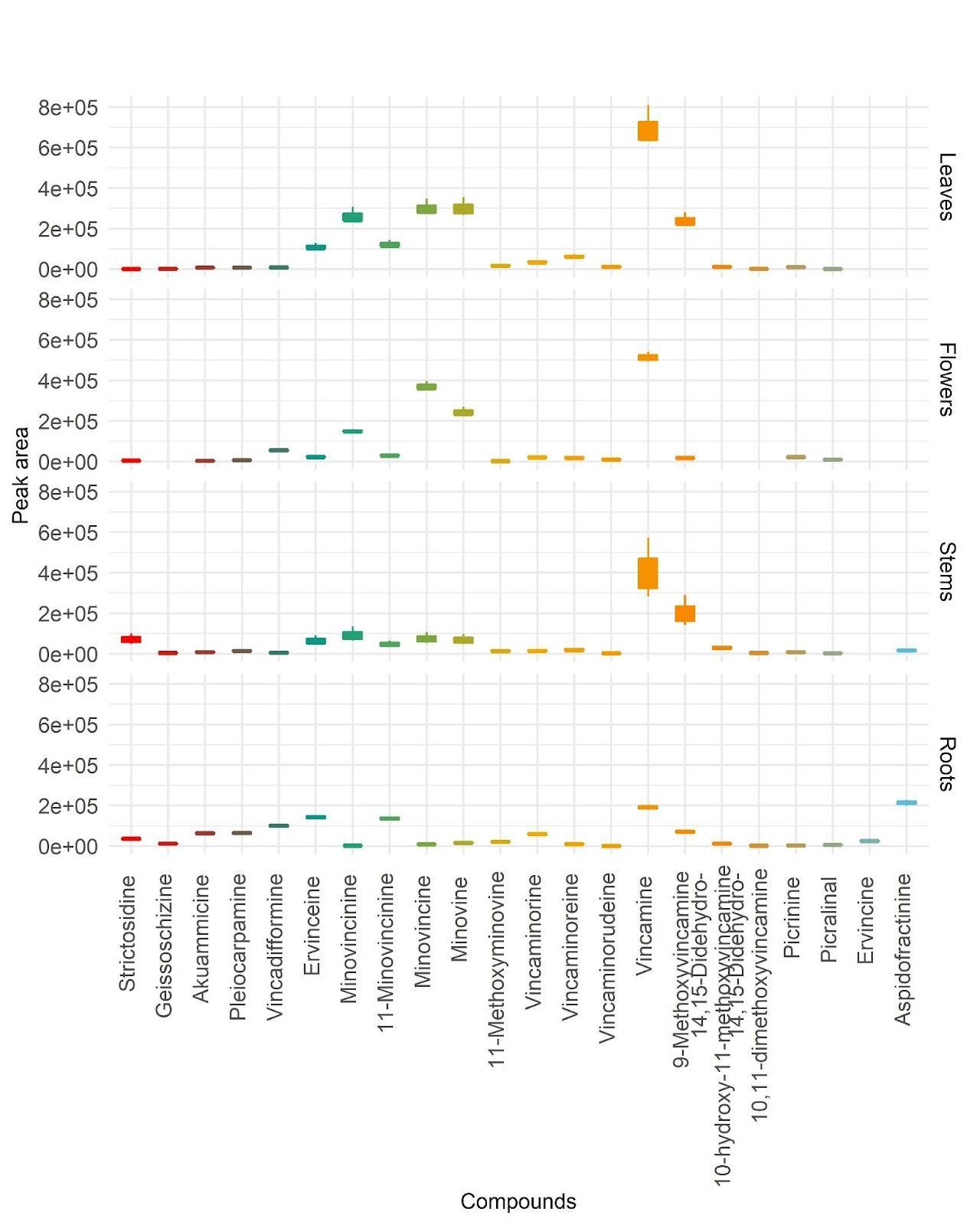


**Figure S5.** UPLC/HRMS peak area comparison of 22 alkaloids compounds identified in *Vinca minor* plant’s organs: leaves, flowers, stems, and roots. Peak area is corrected by dividing the peak area by the ratio:  dry weight of plant/ extract weight obtained.


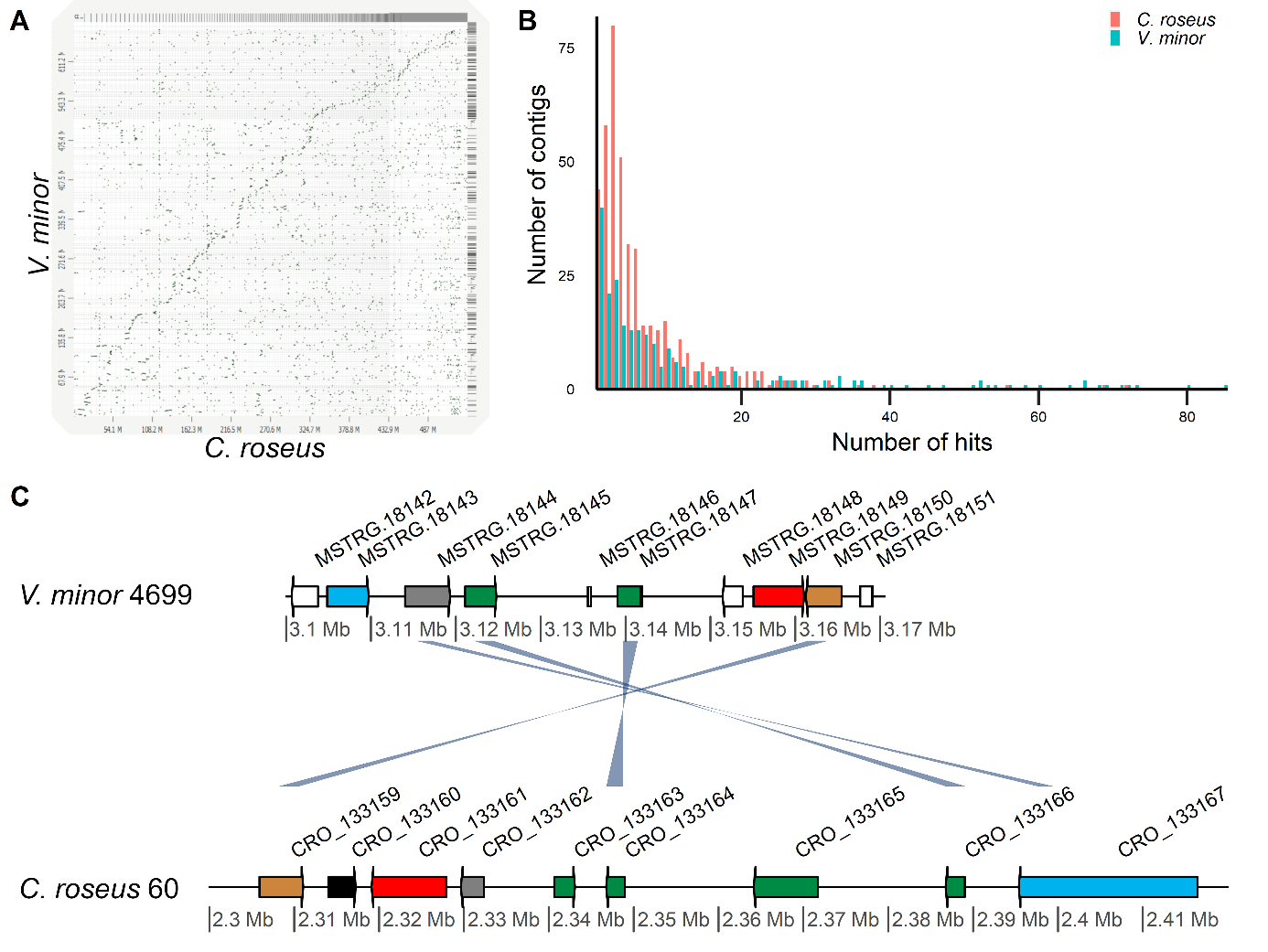


**Figure S6.** Synteny between *V. minor* and *C. roseus* genomes. (A) Genome-wide synteny. (B) Number of contig from *V. minor* (blue) and *C. roseus* (red) with at least one hit. (C) Focus on a region of *C. roseus* contig 60 and *V. minor* contig 4699. Arrows highlight gene orientation. Green: transcription factor (SCARECROW-like), Red: LACCASE, Blue: Sterol transport associated gene, Brown: Metalloprotease, Black: hydrolase, Grey: other function, White: not annotated. Dark blue: antisens match.

**Results: Phytochemical investigation of *Vinca minor.***

UPLC/HRMS with data dependant acquisition of MS2 of the same extracts described above led to the putative identification of 22 monoterpene indole alkaloids (MIA) at three confidence levels, as defined by the Metabolomics Standard Initiative (Chen et al., 2017): Level 1 is by comparison with retention time (RT), accurate mass MS and MS/MS data (accurate mass and fragmentation pattern) of standards, Level 2 is by comparison with MS and MS/MS data with mass spectrum libraries, databases or literature and Level 3 is by comparison of their MS only (Supplemental Table S9). The 22 MIA putatively assigned are Aspidofractinine (**1**) (Vrabec et al., 2022) (Fox Ramos et al., 2019), Minovincinine (**2**) (PLAT et al., 1962), 14,15-Didehydro-10-hydroxy-11-methoxyvincamine (**3**) (Zhang et al., 2007), Minovincine (**4**) (Farahanikia et al., 2011), Vincamine (**5**), Strictosidine (**6**) (Stocicigt & Zenk, 1977), 14,15-Didehydro-10,11-dimethoxyvincamine (**7**) (Zhang et al., 2007), Geissoschizine (**8**)(Stander et al., 2020), 9-Methoxyvincamine (**9**) (Abouzeid et al., 2017), Akuammicine (**10**) (Balsevich et al., 1982), Pleiocarpamine (**11**), Picrinine (**12**) (Grossmann et al., 1973), Ervincine (**13**) (Rakhimov et al., 1967) , Picralinal (**14**), Vincadifformine (**15**) (Mokrý et al., 1963), Minovine (**16**) (Farahanikia et al., 2011), 11-Methoxyminovincinine (16-Methoxyminovincinine) (**17**), Ervinceine (16-Methoxyvincadifformine) (**18**) (Rakhimov et al., 1969), Vincaminorine (**19**), 11-Methoxyminovine (11-Methoxyminovine) (**20**) (Farahanikia et al., 2011), Vincaminorudeine (**21**) (Farahanikia et al., 2011), Vincaminoreine (**22**) (Farahanikia et al., 2011). Moreover, few compounds have not been detected like 9-Hydroxyvincamine, 14,15-Dehydrovincamine, 11-Hydroxyvincadifformine (16-Hydroxyvincadifformine), Echitovenine, Vincarubine, Lochnericine, Tabersonine, Catharanthine, and Hörhammericine.

**References**

Abouzeid, S., Beutling, U., Surup, F., Abdel Bar, F. M., Amer, M. M., Badria, F. A., Yahyazadeh, M., Brönstrup, M., & Selmar, D. (2017). Treatment of Vinca minor Leaves with Methyl Jasmonate Extensively Alters the Pattern and Composition of Indole Alkaloids. *Journal of Natural Products*, *80*(11), 2905–2909. https://doi.org/10.1021/acs.jnatprod.7b00424

Balsevich, J., Constabel, F., & Kurz, W. G. W. (1982). *Alkaloids of Vinca major cv. variegata1* (Vol. 44).

Chambers, M. C., MacLean, B., Burke, R., Amodei, D., Ruderman, D. L., Neumann, S., Gatto, L., Fischer, B., Pratt, B., Egertson, J., Hoff, K., Kessner, D., Tasman, N., Shulman, N., Frewen, B., Baker, T. A., Brusniak, M. Y., Paulse, C., Creasy, D., … Mallick, P. (2012). A cross-platform toolkit for mass spectrometry and proteomics. In *Nature Biotechnology* (Vol. 30, Issue 10, pp. 918–920). https://doi.org/10.1038/nbt.2377

Chen, Q., Lu, X., Guo, X., Guo, Q., & Li, D. (2017). Metabolomics characterization of two apocynaceae plants, catharanthus roseus and vinca minor, using GC-MS and LC-MS methods in combination. *Molecules*, *22*(6). https://doi.org/10.3390/molecules22060997

Farahanikia, B., Akbarzadeh, T., Jahangirzadeh, A., Yassa, N., Reza, M., Ardekani, S., Mirnezami, T., Hadjiakhoondi, A., & Khanavi, M. (2011). *Phytochemical Investigation of Vinca minor Cultivated in Iran*.

Fox Ramos, A. E., le Pogam, P., Fox Alcover, C., Otogo N’Nang, E., Cauchie, G., Hazni, H., Awang, K., Bréard, D., Echavarren, A. M., Frédérich, M., Gaslonde, T., Girardot, M., Grougnet, R., Kirillova, M. S., Kritsanida, M., Lémus, C., le Ray, A. M., Lewin, G., Litaudon, M., … Beniddir, M. A. (2019). Collected mass spectrometry data on monoterpene indole alkaloids from natural product chemistry research. *Scientific Data*, *6*(1). https://doi.org/10.1038/s41597-019-0028-3

Grossmann, E., Sefeovie, P., & Szasz, K. (1973). *PICRININE IN VINCA MINOR* (Vol. 12). Pergamon Press Prmted m England.

Lê, S., Josse, J., & Husson, F. (2008). **FactoMineR** : An *R* Package for Multivariate Analysis. *Journal of Statistical Software*, *25*(1). https://doi.org/10.18637/jss.v025.i01

Mokrý, J., Kompiš, I., Dúbravková, L., & Šefčovič, P. (1963). Vincadifformin und Minovin, zwei weitere racemische Alkaloide ausVinca minor L. *Experientia*, *19*(6), 311. https://doi.org/10.1007/BF02150425

PLAT, M., FELLION, E., le MEN, J., & JANOT, M. (1962). [4 new alkaloids of Vinca minor L.: minovincine, methoxyminovincine, minovincinine and (-) vincadifformine, alkaloids of periwinkles]. *Annales Pharmaceutiques Francaises*, *20*, 899–906.

Pluskal, T., Castillo, S., Villar-Briones, A., & Oresic, M. (2010). MZmine 2: modular framework for processing, visualizing, and analyzing mass spectrometry-based molecular profile data. *BMC Bioinformatics*, *11*, 395. https://doi.org/10.1186/1471-2105-11-395

R Core Team. (2021). R: A Language and Environment for Statistical Computing. In *R Foundation for Statistical Computing*.

Rakhimov, D. A., Malikov, V. M., & Yunusov, S. Y. (1969). The structure of ervinceine. *Chemistry of Natural Compounds*, *5*(4), 280–281.

Rakhimov, D. A., Malikov, V. M., & Yunusov, S. Yu. (1967). Isolation of kopsinilam and ervincine. *Chemistry of Natural Compounds*, *3*(5), 300–301. https://doi.org/10.1007/BF00574649

Stander, E. A., Sepúlveda, L. J., Dugé de Bernonville, T., Carqueijeiro, I., Koudounas, K., Lemos Cruz, P., Besseau, S., Lanoue, A., Papon, N., Giglioli-Guivarc’h, N., Dirks, R., O’Connor, S. E., Atehortùa, L., Oudin, A., & Courdavault, V. (2020). Identifying Genes Involved in Alkaloid Biosynthesis in Vinca minor through Transcriptomics and Gene Co-Expression Analysis. *Biomolecules*, *10*(12), 1595. https://doi.org/10.3390/biom10121595

Stocicigt, B. J., & Zenk, M. H. (1977). *Strictosidine (Isovincoside) : the Key Intermediate in the Biosynthesis of Monoterpenoid Indole Alkaloids*.

Vrabec, R., Maříková, J., Ločárek, M., Korábečný, J., Hulcová, D., Hošťálková, A., Kuneš, J., Chlebek, J., Kučera, T., Hrabinová, M., Jun, D., Soukup, O., Andrisano, V., Jenčo, J., Šafratová, M., Nováková, L., Opletal, L., & Cahlíková, L. (2022). Monoterpene indole alkaloids from Vinca minor L. (Apocynaceae): Identification of new structural scaffold for treatment of Alzheimer’s disease. *Phytochemistry*, *194*. https://doi.org/10.1016/j.phytochem.2021.113017

Zhang, H., Wang, X. N., Lin, L. P., Ding, J., & Yue, J. M. (2007). Indole alkaloids from three species of the Ervatamia Genus: E. officinalis, E. divaricata, and E. divaricata Gouyahua. *Journal of Natural Products*, *70*(1), 54–59. https://doi.org/10.1021/np060344o
